# Flash annealing–engineered wafer-scale relaxor antiferroelectrics for enhanced energy storage performance

**DOI:** 10.1126/sciadv.ady2349

**Published:** 2025-11-14

**Authors:** Yizhuo Li, Kepeng Song, Meixiong Zhu, Xiaoqi Li, Zhaowei Zeng, KangMing Luo, Yuxuan Jiang, Zhe Zhang, Cuihong Li, Yujia Wang, Bing Li, Zhihong Wang, Zhidong Zhang, Weijin Hu

**Affiliations:** ^1^ Shenyang National Laboratory for Materials Science, Institute of Metal Research, Chinese Academy of Sciences, Shenyang 110016, People’s Republic of China.; ^2^ Electron Microscopy Center, School of Chemistry and Chemical Engineering, Shandong University, Jinan 250100, People’s Republic of China.; ^3^ School of Materials Science and Engineering, University of Science and Technology of China, Shenyang 110016, People’s Republic of China.; ^4^ Electronic Materials Research Laboratory, School of Electronic Science and Engineering, Xi’an Jiaotong University, Xi’an 710049, People’s Republic of China.

## Abstract

Dielectric capacitors are essential for energy storage systems because of their high-power density and fast operation speed. However, optimizing energy storage density with concurrent thermal stability remains a substantial challenge. Here, we develop a flash annealing process with ultrafast heating and cooling rates of 1000°C per second, which facilitates the rapid crystallization of PbZrO_3_ film within a mere second, while locking its high-temperature microstructure to room temperature. This produces compact films with subgrain boundary fractions of 36%, nanodomains of several nanometers, and negligible lead volatilization. These contribute to relaxor antiferroelectric film with a high breakdown strength (4800 kilovolts per centimeter) and large polarization (70 coulombs per square centimeter). Consequently, we have achieved a high energy storage density of 63.5 joules per cubic meter and outstanding thermal stability with performance degradation less than 3% up to 250°C. Our approach is extendable to ferroelectrics like Pb(Zr_0.52_Ti_0.48_)O_3_ and on wafer scale, providing on-chip nonlinear dielectric energy storage solutions with industrial scalability.

## Teaser

Flash annealing ultrafast crystallizes PbZrO_3_, yielding a relaxor antiferroelectric state that boosts energy storage performance.

## INTRODUCTION

Dielectric capacitors store electrical energy through electric displacement, owning distinct merits including high power density and ultrafast charge/discharge speed, are promising for applications in modern energy storage devices such as consumer electronics, high-power lasers, and radar systems (*1*–*3*). Nevertheless, compared to chemical energy sources like Li-ion batteries and solid oxide fuel cells, they have a relatively low energy density (*4*). Consequently, extensive efforts have been devoted to enhance the energy density of dielectric materials with high efficiency, in response to the ongoing trend of device miniaturization in electronic systems (*5*–*7*). The device performance is evaluated by the recoverable energy density *U*_e_ = $\int_{P_{r}}^{P_{m}}\mathrm{EdP}$ and the energy storage efficiency ƞ *= U*_e_/(*U*_e_ + *U*_loss_), where *P*_m_ and *P*_r_ are the maximum and remanent polarization, respectively (fig. S1). *U*_loss_ is determined by the hysteresis of *P*-*E* loop, which not only limits *U*_e_, but also degrades η, resulting in energy dissipation problem that deteriorates the device thermal stability. Hence, a large *P*_m_ with a minor hysteresis is vital for achieving a good energy storage performance. In this regard, nonlinear dielectrics such as ferroelectrics (FE) and antiferroelectrics (AFE) are competitive because of large *P*_m_. However, they usually suffer from apparent loss associated with the switching of the FE domains or the first-order field-induced AFE-FE phase transition (*8*).

To address this issue, the main idea has been to develop relaxor FE (RFE) or relaxor AFE (RAFE) by reducing the domain size from micrometer to nanometer scale, which can weaken the domain intercoupling and break the long range–ordered domains into short range–ordered nanodomains (*9*). This process lowers the switching barrier and smears out the hysteresis, thereby enhancing both *U*_e_ and η. On the basis of this framework, approaches including multiphase composition (*10*), solid solution (*11*), chemical doping (*5*), and defect engineering (*12*) have been put forth to facilitate the design of RFE or RAFE materials. Representative examples include constructing room temperature RAFE in AgNbO_3_ via solid solution with AgTaO_3_ to obtain a *U*_e_ of 6.3 J/cm^3^ and η of 90% (*11*), creating a superparaelectric state in BiFeO_3_-BaTiO_3_ RFE system by chemical doping and get a *U*_e_ of 152 J/cm^3^ and η of 77% (*5*), or achieving a *U*_e_ of 133 J/cm^3^ and η of 75% in 0.68Pb(Mg_1/3_Nb_2/3_)O_3_-0.32PbTiO_3_ RFE film by introducing point defects through high-energy ion bombardment (*12*). In addition to the nanodomain design, grain refinement has also been explored to augment energy storage capabilities, recognizing that the reduction in grain size (*d*) can enhance the breakdown field (*E*_b_ ∝*d*^-α^, where α *~* 0.2 to 0.4) across diverse dielectrics (*13*, *14*). Implementing methods include chemical doping (*15*), multiphase composition (*16*), high-entropy stabilization (*17*), and hot-pressing treatment (*18*). However, these approaches are commonly associated with several drawbacks: The requirement for exact stoichiometry and precise control of interface microstructures necessitates intricate fabrication procedures, which not only elevate production costs but also complicate manufacturing processes, creating substantial barriers to large-scale production. To overcome these challenges, there is an urgent need for in-depth research and technological innovation.

**Fig. 1.**
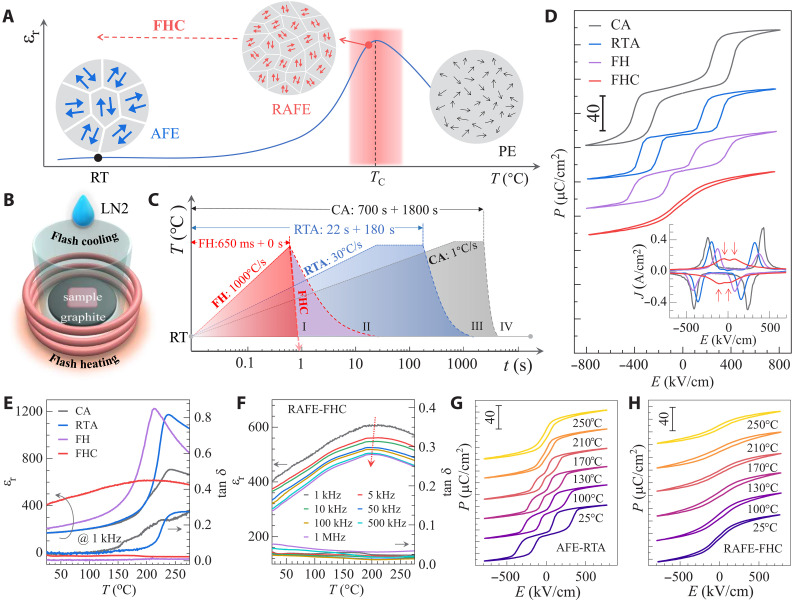
Fundamental design principles of relaxor antiferroelectric materials. (**A**) The temperature-dependent dielectric permittivity (ε_r_) of an AFE material depicted alongside representative schematic domain patterns at various temperatures. (**B**) Illustration of the experimental setup for FHC, capable of achieving rates up to 1000°C/s, enabling the synthesis of RAFE materials within a single second. (**C**) A comparison of diverse heat treatment protocols, with treatment times spanning from less than 1 s for FHC to more than 1000 s for CA. (**D**) *P-E* hysteresis loops for PZO films produced using different annealing techniques. The inset shows the corresponding switching current (*J-E*) curves. The red arrows indicate the peak-current positions for film processed by FHC. (**E**) Temperature-dependent dielectric and loss for various films. (**F**) Temperature-dependent dielectric for RAFE film at different frequencies. (**G** and **H**) *P-E* loops at various temperatures for AFE (G) and RAFE (H) films.

We noticed that FEs or AFEs display relaxor characteristics with a large amount of nanodomains near Curie temperature (*T*_c_) (Fig. 1A) because of the flattened domain-switching pathway with reduced energy barrier at high temperature. If one can freeze the high-temperature nanodomains down to room temperature (Fig. 1A), a slim *P-E* loop with a large *P*_m_ is expected to sustain, leading to large *U*_e_ and η. Inspired by the conventional quenching techniques used in structural materials (*19*), we believe that an intuitive approach to achieve this goal involves rapid crystallization succeeded by quenching, which tends to inhibit the normal grain growth and the emergence of long-range ordered domains, thereby retaining the nanodomains even at room temperature. To conduct this process, we develop a flash heating and cooling (FHC) method that features both high heating and cooling rate (~1000°C/s), enabling the ultrafast sintering within less than 1 s. Specifically, we put the films together with a graphite substrate into a copper coil that can rapidly heat the film through electromagnetic induction and thermal conduction (Fig. 1B). In addition, the noncontact heating feature allows us to execute the flash cooling within 1 s by immersing the films into the liquid nitrogen (LN2).

## RESULTS

### FHC-engineered relaxor antiferroelectric characters

To test the feasibility of FHC, we synthesized PbZrO_3_ (PZO) AFE films, which are extensively considered for energy storage applications. We commenced by spin-coating PZO amorphous films via chemical solution deposition (CSD), followed by FHC treatment (Fig. 1C and table S1). For comparison, we conducted a series of control treatments, including conventional muffle furnace annealing (CA), rapid thermal annealing (RTA), and flash heating (FH) with subsequent air cooling. The heating rates differ substantially, with 1°C/s for CA, 30°C/s for RTA, and a remarkable 1000°C/s for both FH and FHC, enabling a thorough examination of heating rates on material properties over three orders of magnitude. The cooling process involves air cooling for CA, RTA within a time of ~1000 s, FH within 30 s, and FHC within a mere 1 s (fig. S2). Figure 1D illustrates that PZO film produced by FHC process exhibits a slim *P-E* loop with negligible hysteresis, a characteristic distinct from the typical AFE double-hysteresis loops for films fabricated by other methods. The corresponding switching current curves (*J-E,* inset of Fig. 1D) reveal four peaks that are smaller in intensity and broad in width for the FHC film, demonstrating its RAFE characteristics. We also performed similar FHC process by varying the heating rates (fig. S3). Substantial relaxor behavior emerges when the heating rate is up to ~500°C/s. These findings suggest that both FH (i.e., within 650 ms) and LN2 quenching (i.e., <1 s) are critical for developing the RAFE state. The evolution from AFE to RAFE has also been reflected in the dielectric (ε_r_) spectrum (Fig. 1, E and F). ε_r_ at 1 kHz and ambient temperature are 167, 168, 206, and 404 for films processed by CA, RTA, FH, and FHC, respectively, with the RAFE-FHC film achieving a 2.5-fold enhancement in ε_r_ than that of AFE film. This increase in ε_r_ aligns well with Fig. 1A, wherein the RAFE phase exhibits smaller domains, making them easier to be rotated by the electric field. With increasing the temperature, AFE treated by CA, RTA, and FH exhibit a typical sharp dielectric peak at *T*_c_ ~ 220–240°C (see also fig. S4). Conversely, FHC film exhibits a pronounced broadening of ε_r_ over a wide temperature range, indicating a profound diffuse phase transition between RAFE and paraelectric states. The relaxor diffuseness factor γ, as derived from the modified Curie-Weiss law (*20*), is ~2.0 for FHC film, notably higher than AFE phase treated by other methods (γ = 1.5, 1.3, and 1.7 for CA, RTA, and FH films, respectively) (fig. S5). The FHC-induced relaxor behavior exhibits a minimal frequency dispersion, with *T*_c_ differing by only ~3 K between 1 kHz and 1 MHz (Fig. 1F). This *T*_c_ shift is smaller than other PZO RAFEs engineered by chemical doping such as Li^+^–Al^3+^ codoping (~10 K) (*21*), and by Al_2_O_3_ interface engineering (~6 K) (*22*). Consistently, the dielectric loss (tan δ, fig. S6) also exhibits minimal frequency dispersion, maintaining small values between 0.01 and 0.05 across all frequencies. This exceptionally weak frequency dependence is attributed to the paraelectric-like crystal structure and robust grain boundaries produced by FHC process, as detailed in subsequent sections. Moreover, the relaxor *P-E* loops of FHC film remain stable from 25° to 250°C (Fig. 1H), demonstrating a remarkable thermal stability. In sharp contrast, *P-E* loops of AFE film gradually transformed into the RAFE state with increasing temperature, showing strong thermal dependence (Fig. 1G). This contrast is further evidenced in current switching curves (fig. S7), where FHC films display a smaller AFE-FE transition field that is weakly dependent on temperature. Such a weak frequency and temperature dispersion of ε_r_ and *P-E* loop for FHC film suggests its suitability for applications requiring stringent frequency and thermal stability, including high-precision capacitors and energy storage devices.

### The microscopic origin of FHC-induced RAFE

**Fig. 2.**
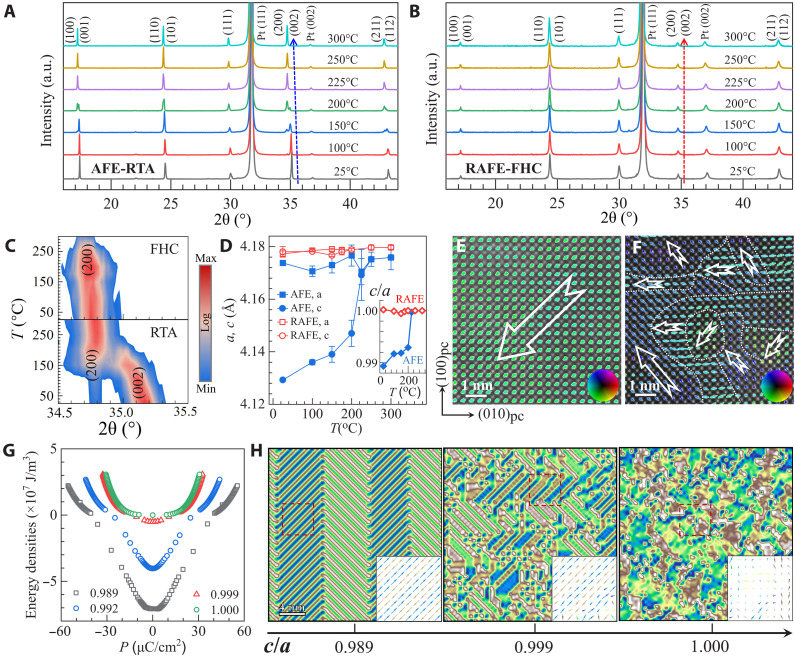
The crystal structural characteristics of AFE and RAFE films. (**A** and **B**) XRD patterns at various temperatures for PZO films treated by RTA (A) and FHC (B). (**C**) The detailed view of (200)/(002) diffraction peaks. (**D**) Temperature dependent lattice constants. The inset represents the *c*/*a* ratio as a function of temperature. (**E** and **F**) HAADF-STEM images for AFE-RTA (E) and RAFE-FHC (F) films. The arrows at each ion show A-site cation displacement vectors in each unit cell. The orange arrows mark the domains of different orientations. (**G** and **H**) Free energy profiles (G) and domain patterns (H) with the increase of *c*/*a* ratio predicted by phase-field simulations. Insets in (H) show enlarged polarization vector fields from the red dashed boxes, offering a clearer view of local polarization distribution. a.u., arbitrary units.

To gain insights into the structure origin of this robust RAFE state, we performed high-resolution x-ray diffraction (HRXRD; fig. S8) on PZO films by using synchrotron x-ray source at 10 keV. While all films display similar diffraction patterns, there is a systematic shift of diffraction peaks to lower angles with increasing the heating rates from CA to RTA, and further to FH and FHC, implying the expansion in lattice constants and cell volume. Consequently, we chose the AFE-RTA and RAFE-FHC films to investigate their temperature-dependent HRXRD profiles, as depicted in Fig. 2 (A and B). As indicated by the dashed arrows, the diffraction peaks of AFE-RTA film shift toward lower angles as the temperature rises, whereas, they remain unchanged for RAFE-FHC film. In addition, the expanded view of the diffraction profile around the (200) reflection (Fig. 2C) and at other planes—including (100), (110), and (211) (fig. S9)—reveals distinct peak splitting (~0.4° at 25°C) in the AFE-RTA film, attributed to tetragonal distortion. In contrast, no peak splitting appears in RAFE-FHC film. This structure difference has been quantitatively reflected in their lattice constant values (Fig. 2D) as derived from HRXRD. Notably, the lattice constants *a* and *c* of AFE film (Tetragonal, space group P4mm) are clearly different at room temperature, giving rise to a *c*/*a* ratio of ~0.9893. The sharp increase of *c* near *T*_c_ of ~230°C makes them eventually merge into the paraelectric state (Cubic, space group Pm-3 m). Consequently, the RTA film exhibits notable anisotropic thermal expansion in its AFE state. Such anisotropy, as has also been observed in PZO bulk ceramics (*23*), is characteristic of materials with reduced crystal symmetry. Meanwhile, the lattice constants *a* and *c* of RAFE film are nearly equal and identical to those of the paraelectric phase. As a result, RAFE film exhibits a minor *c*/*a* ratio expansion (from 0.999 to 1), in sharp contrast with that of AFE film (from 0.989 to 1) across *T*_c_ (inset of Fig. 2D). This comparison clearly suggests that FHC treatment can effectively preserve the lattice configuration of the high-temperature state down to room temperature. The paraelectric-like RAFE state with minor structural distortion enables the formation of nanodomain with size shrinking down to 1 to 3 nm, as evidenced by the high-angle annular dark-field scanning transmission electron microscopy (HAADF-STEM) image presented in Fig. 2F. In contrast, AFE-RTA film exhibits long range–ordered domains with large size of more than ~10 nm (Fig. 2E). This strong sensitivity of AFE/RAFE configuration on the structure distortion *c*/*a* has been further revealed by the phase-field simulation (Fig. 2, G and H). We found that the free energy barrier between the nonpolar AFE state and PE states reduces substantially from 7.20 × 10^7^ to 0.02 × 10^7^ J/m^3^ as the *c*/*a* ratio increases from 0.989 to 1.000, leading to the transformation of long-range ordered domain to the disordered nanodomain with reduced domain size (Fig. 2H). The decreased barrier facilitates the phase transition, thereby reducing the field required to achieve the saturation polarization and effectively minimizing energy losses in the hysteresis loops. Correspondingly, the double-hysteresis *P-E* loops change into the slim relaxor loops as the *c*/*a* increases (fig. S10), a change that aligns well with the experimental observations (Fig. 1D).

**Fig. 3.**
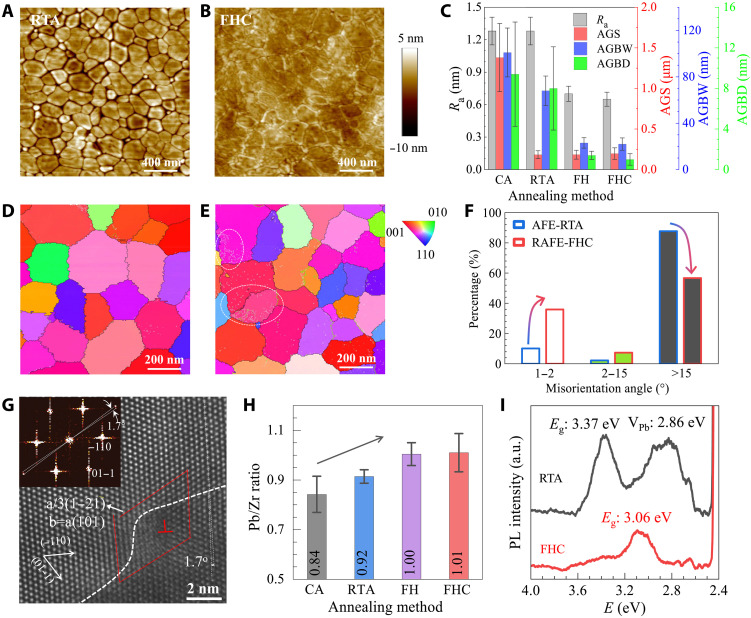
The microstructure characteristics. (**A** and **B**) Topographic images for RTA-AFE (A) and FHC-RAFE (B) films. (**C**) Summary of surface roughness (*R*_a_), average grain size (AGS), average grain boundary depth (AGBW), and width (AGBD) for various PZO films. (**D** and **E**) The IPF and GB situations for RTA-AFE (D) and FHC-RAFE films (E). (**F**) The misorientation angle statics derived from (D) and (E). (**G**) HAADF image of a sub-GB in RAFE film with a misorientation angle of 1.7^o^. Inset, the fast Fourier transform diffraction patterns. (**H**) Pb/Zr atomic ratio statics derived from EDS analysis. (**I**) PL spectrums of RTA-AFE and FHC-RAFE films.

In addition to the nanodomain engineering, FHC also has prominent impact on the grain distributions, as revealed by surface images shown in Fig. 3 (A and B) and fig. S11. Take RTA-AFE (Fig. 3A) and FHC-RAFE (Fig. 3B) films as an example: Both share similar grain size distribution with average grain size of 183 and 197 nm, respectively. However, RAFE film has a rather smooth surface with a small average roughness *R*_a_ of ~0.65 nm compared to that of RTA-AFE film (*R*_a_ ~ 1.28 nm) because of the denser packed crystal grains characterized by a narrow grain boundary width (GBW) and shallow GB depth (GBD). The average GBW and GBD are ~22 and 1.0 nm for FHC-RAFE film, whereas they increase to ~68 and ~8 nm for RTA-AFE film (fig. S11, D and E). This noticeable difference is also clearly visible in the TEM images (fig. S12). We represent these metric parameters in Fig. 3C, highlighting that both the FH and FHC processes substantially improve the grain compactness and surface smoothness, thanks to the ultrafast crystallization that effectively avoids the grain contraction during the cooling process. To reveal more details of GB, we further performed transmission Kikuchi diffraction (TKD), and obtained the inverse pole figure (IPF; Fig. 3, D and E) mapping for FHC-RAFE and RTA-AFE films. The IPF analysis reveals that RAFE film has more subgrain boundaries (sub-GB, indicated by white dashed circles) characterized by misorientation angle of 1° to 2° than that of AFE film. As summarized in Fig. 3F, the fraction of sub-GB increases from 10.1% for AFE film to 35.9% for RAFE film. In contrast, the normal large-angle GB (misorientation angle >15°) decreases from 87.7% for AFE film to 56.7% for RAFE film. A typical sub-GB, marked by the dashed line, is further visualized by the STEM image (Fig. 3G), which is associated to a perfect dislocation with a Burgers vector of **a**[101]. By performing fast Fourier transform on the STEM image, we observed two sets of barely distinguishable diffraction spots (inset of Fig. 3G), and the misorientation angle of this sub-GB is determined to be ~1.7°. Moreover, we identified a characteristic GB with a larger misorientation angle of ~4.5°, which is associated with a network of dislocations (fig. S13). This finding is consistent with the consensus that the formation of GB/sub-GB is a consequence of dislocation pile-up at the boundary (*24*). We attribute the formation of sub-GB to the FHC process, where flash cooling within 1 s suppresses grain growth and raises the dislocation density within grains, promoting sub-GBs formation via the rearrangement and intersections of dislocations during the cooling process. The cumulative effect of crystal misalignment and misfit dislocation at sub-GBs impedes long-range domain ordering, promoting nanodomain formation and stabilizing the RAFE phase. Concurrently, sub-GBs induce notable internal stress, as qualified by kernel average misorientation maps (fig. S14) showing larger intragrain misorientation angles at GBs and sub-GBs. This confirms intensified lattice distortion and residual stress that can be further quantified via Williasson-Hall analysis (*25*) of XRD peak broadening (fig. S15). Residual stress increases from 0.036% for CA, 0.13% for RTA, 0.12% for FH, and ultimately to 0.18% for FHC, demonstrating how thermal processing rates critically tailor PZO film microstructures to achieve RAFE properties.

Furthermore, FHC effectively suppresses Pb volatilization, a common issue in conventional long-time annealing processes. This suppression is corroborated by EDS analysis (Fig. 3H), which shows a systematic increase in Pb content with reduced processing time: The Pb: Zr atomic ratio rises from 0.84 ± 0.07 (CA) to 1.01 ± 0.08 (FHC). Additional EDS mapping (fig. S16) further indicates that Pb deficiency primarily localizes at grain boundaries. This Pb deficiency is likely the root cause of the subtle dielectric kink observed in CA films (Fig. 1E). The stoichiometric Pb: Zr ratio in RAFE films is further supported by photoluminescence (PL) spectra (Fig. 3I). The characteristic PL peak attributed to Pb vacancies (V_Pb_), observed at ~0.5 eV away from the band edge in RTA film, is absent in the RAFE-FHC film. The position of this PL peak aligns well with energy levels of V_Pb_ predicted from first-principles calculations (~0.7 eV; fig. S17). Concurrently, the bandgap of RAFE film (~3.06 eV) is ~0.3 eV smaller than that of AFE film (~3.37 eV), which is attributed to the combined effect of lattice constants and V_Pb_ concentrations, as supported by the bandgap values summarized in table S2.

### Electric properties and energy storage performance

**Fig. 4.**
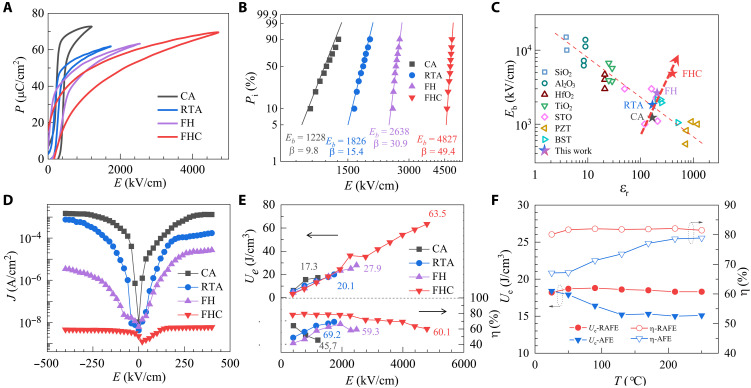
Polarization, electric, and energy storage properties. (**A**) *P-E* hysteresis loops. (**B**) Two-parameter Weibull distribution analysis of the characteristic breakdown fields *E*_b_. (**C**) *E*_b_ versus dielectric constant for typical dielectric materials. (**D** and **E**) Leakage current (D) and energy density and efficiency (E) as a function of electric field for PZO films treated by different annealing methods. (**F**) Temperature dependent energy storage performance at 1500 kV/cm for AFE and RAFE films.

To evaluate the energy-storage performance, we measured *P-E* loops by applying large electric field (Fig. 4A). Compared to other films, RAFE-FHC film sustains a larger electric field while maintaining a big *P*_m_ of ~70 μC/cm^2^*.* As revealed by the *P-E* loops under increasing electric fields (fig. S18), this large polarization originates from the field-induced RAFE-FE transition. The Weibull distribution plots (Fig. 4B) show that the RAFE-FHC film has an excellent breakdown electric field (*E*_b_) of 4827 kV/cm, much higher than that of AFE films treated by other processes (CA: 1228 kV/cm; RTA: 1826 kV/cm; FH: 2638 kV/cm). Meanwhile, the Weibull modulus β increases from 9.8 for AFE-CA film to 49.4 for RAFE-FHC film, suggesting the improved reliability and uniformity by FHC process. It is well known that there is an intrinsic trade-off between ε_r_ and *E*_b_, with a larger ε_r_ that usually signifies a lower *E*_b_ for dielectric materials (Fig. 4C) (*14*, *26*, *27*). Our RAFE-PZO film has both good ε_r_ and *E*_b_. We ascribe this substantial improvement in *E*_b_ to the FHC-induced compact grains, subgrain boundaries, and pronounced suppression of V_Pb_. These features efficiently block leakage paths, enhance the electron scattering, and reduce the carrier concentrations, drastically diminishing the leakage current to ~10^−8^ A/cm^2^ under a dc field of 400 kV/cm for the RAFE-FHC film—five orders of magnitude smaller than that of the AFE-CA film (~2 × 10^−3^ A/cm^2^) (Fig. 4D).

The remarkable combination of high *E*_b_, large *P*_m_, and slender hysteresis result in a high *U*_e_ of 63.5 J/cm^3^ for RAFE-FHC film, which is two to four times that of AFE films treated by CA (~17.3 J/cm^3^), RTA (~20.1 J/cm^3^), and FH (~27.9 J/cm^3^) (Fig. 4E). We also found a maximum energy storage efficiency η of ~80% at an *E* of <2000 kV/cm, which remains at level of ~60% at *E*_b_ for the RAFE-FHC film. As revealed in fig. S19, this substantial performance enhancement primarily stems from microstructure engineering (subgrains and nanodomains), with Pb stoichiometry providing secondary gains. Notably, the energy storage performance maintains over a broad temperature range up to 250°C (Fig. 4F). This excellent thermal stability guarantees proper functioning of the device under extreme temperature conditions, including the hybrid electric vehicles (<140°C) and the harsh environments of underground oil/gas exploration (<200°C) (*28*, *29*). We ascribe it to the stable relaxor behavior of RAFE state (Fig. 1H). The FHC process effectively preserves the high-temperature structure, endowing the film with temperature-insensitive characteristics. In addition, the excellent insulating nature with minor *V*_Pb_ defects mitigate the thermal activation of carriers, thereby minimizing the conduction losses at elevated temperatures (Fig. 1E). Last, both RTA and FHC films maintain stable energy storage performance up to 10^7^ cycles without breakdown (fig. S20). This endurance is comparable to state-of-the-art values reported for thin-film capacitors based on PZO (~10^7^) (*30*) and other materials such as BiFeO_3_-BaTiO_3_ solid solutions (~10^8^) (*5*). We summarize the metrics of pristine PZO films fabricated using diverse techniques including magnetic sputtering, pulsed laser deposition, CSD processed with microwave radiation, among others, in Table 1 (*30*–*34*). Our RAFE-FHC capacitor device boasts the best performance, featuring exceptional thermal stability with *U*_e_ and η degradation of less than 3% up to 250°C*.* The flash annealing treatment, requiring less than 1 s, is at least two orders of magnitude faster than other methods, suggesting its high efficiency in producing RAFE-PZO films.

**Table 1.** Energy storage performance of PZO films fabricated by various methods.

Method	State	Ann. time (s)	*P_m_* (μC/cm^2^)	*E_b_* (kV/cm)	*U_e_* (J/cm^3^)	η (%)	Thermal stability (Δ*U_e_* , Δη%)	Ref.
Magnetron sputtering	AFE	1800	45	700	12.5	-	-	(*31*)
Pulsed laser deposition (PLD)	AFE	3600	50	2000	46.0	63	24 to 250°C (≤47, ≤38%)	(*32*)
PLD and ion implantation	RFE	3000	60	4493	62.3	60	25 to 125°C (≤5, ≤5%)	(*33*)
CSD and RTA	AFE	180	45	664	16.6	50	25 to 140°C (≤41, ≤20%)	(*30*)
CSD and microwave radiation	AFE	180	56	765	14.8	59	-	(*34*)
CSD and FHC	RAFE	1	70	4827	63.5	60	25 to 250°C (≤3, ≤3%)	This work

**Fig. 5.**
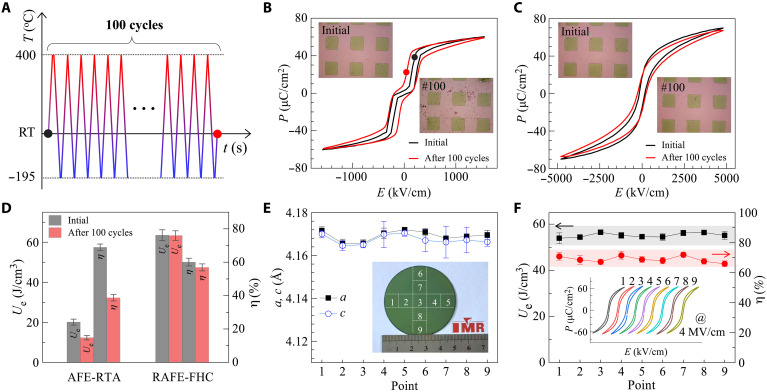
Thermal cycling reliability and scalability measurements. (**A**) Protocol for thermal cycling test. (**B** and **C**) *P-E* loops of the pristine state and state after 100 thermal cycles for (B) RTA-AFE film and (C) FHC-RAFE film. Insets are the corresponding optical images of the films. (**D**) Energy storage performance in the pristine state and that after 100 thermal cycles. (**E**) Lattice constants on nine typical positions across a 5.08 cm PZO film upon FHC treatment. Inset represents the optical image of the 5.08 cm film with the numbers indicating the locations of the measurements. (**F**) *U*_e_ and η on the nine typical positions at 4000 kV/cm. Inset provides the corresponding *P*-*E* loops.

In addition, the minor temperature-dependent lattice variation of the FHC-RAFE film, enabling it to withstand extreme thermal cycling test from LN2 to 400°C, as shown in Fig. 5A. After 100 thermal cycles, the *P-E* loop of RTA-AFE capacitor broadens substantially (Fig. 5B), while the FHC-RAFE capacitor exhibits only subtle changes (Fig. 5C). This is consistent with the optical images (inset of Fig. 5, B and C): The RTA-AFE film has more pores and structural defects after cycling, whereas the FHC-RAFE film maintains its structural integrity. Consequently, the FHC-RAFE capacitor experiences minor degradation with energy storage density dropping from 63.5 to 63.3 J/cm^3^ and efficiency from 60.1 to 56.9%, much smaller than that of RTA-AFE capacitor with energy density declining from 20.1 to 12.3 J/cm^3^ and efficiency dropping from 69 to 38.7% (Fig. 5D). The FHC treatment is compatible with large-scale production. As represented in inset of Fig. 5E, we fabricated an RAFE film on a 2-inch (5.08 cm) platinized silicon wafer. XRD results from nine typical positions show lattice constants *a* and *c* consistent with the RAFE state and a minor variation within 0.08% (Fig. 5E), indicating exceptional structural homogeneity. The film also achieves uniform energy storage characteristics, with an *U*_e_ of 54.5 ± 2.7 J/cm^3^ and an η of 69.1 ± 4.9% (Fig. 5F). The excellent tolerance to extreme thermal cycling renders FHC-RAFE highly suitable for applications involving frequent temperature changes, such as in the aerospace field, electric vehicles, and 5G communication base stations where capacitors encounter complex thermal environments. Moreover, the results underscore the FHC’s advantage in high-throughput manufacturing of RAFE films and microcapacitors, providing on-chip energy storage solutions with industrial scalability.

## DISCUSSION

We have developed a flash annealing technique characterized by ultrafast heating and cooling rates reaching 1000°C/s, which enables the rapid crystallization of PZO films within a mere second, effectively locking the high-temperature nanodomain configurations down to room temperature and giving rise to a relaxor antiferroelectric state with enhanced energy storage performance including the energy storage density and the excellent thermal stability. Note that this ultrafast crystallization process is also compatible with Pt as the top electrode, demonstrated by RAFE like *P-E* loop of a typical La_0.67_Sr_0.33_MnO_3_/PZO/Pt capacitor treated by FHC process (fig. S21), confirming viability for device integration. The nanodomain and subgrain design by FHC can also be used on typical FEs like Pb(Zr_0.52_Ti_0.48_)O_3_, transforming it into the RFE state with *U*_e_ being enhanced by more than fivefold from 10.7 to 57.8 J/cm^3^, while keeping η at above 62.3% (fig. S22). This demonstrates its versatility in promoting the relaxor domain state and the energy storage performance of nonlinear dielectrics. More generally, the strategy is applicable to systems where nanodomain and subgrain engineering are critical in determining the specifical functionalities, such as dielectric and piezoelectric materials.

## MATERIALS AND METHODS

### Amorphous thin-films fabrication

We grew amorphous PZO thin films on Pt/Ti/SiO_2_/Si substrates via CSD. First, we dissolved stoichiometric Pb(CH_3_COO)_2_·3H_2_O in 2-methoxyethanol at 120°C for 240 min, adding a 10% lead excess to compensate for annealing loss. After cooling to room temperature, we introduced Zr(OCH_2_CH_2_CH_3_)_4_ and stirred the solution for 60 min. We then adjusted the concentration to 0.4 M by diluting with 2-methoxyethanol, matching our prior work (*35*). After aging the solution for 24 hours, we spin-coated it onto substrates at 3000 rpm for 30 s, pyrolyzed it at 450°C for 5 min, and repeated this cycle to achieve the desired thickness (fig. S23). To prevent final lead loss, we applied a PbO capping layer via spin coating and then performed various thermal treatments.

### Setup for the FH and FHC processes

We assembled the electromagnetic induction heating equipment with an induction unit (Haidewei-HDWG), program control system, sample stage, and infrared pyrometer (INPTEK-IS30). We designed the sample stage as a 30-mm-diameter high-purity graphite disk placed in a quartz crucible and suspended an LN2 nozzle above it. The super high-frequency induction unit allows current adjustment (0 to 10 A) and frequency tuning (50 to 400 kHz). We coupled the infrared pyrometer (±0.5% reading +2°C accuracy, 5-ms response) to an electromagnetic valve at the LN2 outlet, enabling millisecond quenching upon reaching target temperatures. Our system controls heating rates up to a maximum value of 1500°C/s and subsequent temperature holds or cooling in air (FH) or LN2 (FHC).

### The CA and RTA processes

For CA, we heated the amorphous PZO film in a muffle furnace to 700°C at 1°C/s, held it for 30 min, and then cooled it in air to room temperature. For RTA, we rapidly heated the film to 650°C at 30°C/s in an RTA furnace, held it for 180 s, and then cooled it in air to room temperature.

### Structural characterizations

We investigated the phase composition and stress changes in PZO thin films at various temperatures using HRXRD with a 10-keV synchrotron source at Beamline BL02U2 of the Shanghai Synchrotron Radiation Facility. We analyzed atomic structures using probe-corrected STEM (Thermo Fisher Scientific, Spectra 300, 200 kV) equipped with a Gatan Continuum 1065 detector, setting HAADF collection angles to 50 to 200 mrad and convergence angles to 21.6 mrad. We prepared cross-sectional samples using the focused ion beam (FIB) (Thermo Fisher Scientific, Helios G4), achieving 60-pm spatial resolution. To identify local dipoles, we tracked Pb^2+^ displacement vectors (strong contrast) relative to four nearest Zr^4+^ centers (weak contrast) and performed two-dimensional (2D) Gaussian fitting using custom MATLAB code (*36*).

We characterized surface morphologies by atomic force microscopy (AFM, Bruker Multimode-8) in tapping mode using Pt/Ir-coated Si cantilevers and analyzed grain size distributions via the intercept method (Heyn method, Nano Measurer). This method calculates grain dimensions as the total intercept length of random test lines divided by the number of GB intersections (fig. S24). We quantified GB width/depth using NanoScope Analysis, performing line scans across 100 randomly selected GBs in AFM images. We determined grain orientations/microstructures via TKD using a scanning electron microscope (Gemini SEM 300) outfitted with an EBSD detector (Oxford Symmetry S2) and analyzed data using Aztec Crystal 2.1. We measured composition using energy-dispersive x-ray PL spectrums using a fluorometer (Hitachi F-4500) with a 250-nm excitation wavelength.

### Device fabrication and electric characterizations

For electrical measurements, we fabricated capacitor devices with Pt top electrodes (50 μm by 50 μm) deposited by a dc magnetron sputtering (JGP560CIV) and patterned by a standard photolithography and lift-off processes (URE-2000/35). We performed *P-E* loops, dielectric spectrums, and leakage current using an FE tester (Precision Multiferroic, Radiant Tech), an LCR meter (Tonghui TH2838), and a multisource meter (Keithley 2450) equipped on a high-temperature probe station (Gogo Instruments Tec., LRT-001-D4).

### Weibull distribution analysis

We calculated the value of *E*_b_ via the following formula (*37*)

$$X_{i}=\text{ln}E_{i}$$ (1)

$$Y_{i}=\text{ln}\text{ln}\frac{1}{1-P_{i}}$$ (2)

$$P_{i}=\frac{i}{n+1}$$ (3)

where *n* = 10 represents the number of devices, *E*_*i*_ denotes the electric breakdown strength of each sample arranged in ascending order (*E*_1_ ≤ *E*_2_ ≤ *E*_3_... ≤ *E*_n_), and *P*_*i*_ represents the probability of dielectric breakdown. By using the two-parameter Weibull distribution function, we established a linear relationship between *X*_*i*_ and *Y*_*i*_ and then extracted the average *E*_b_ from the point where the fitting line intersects with the horizontal axis at *Y*_*i*_ = 0. Subsequently, we obtained *P*_s_, *P*_r_, and hysteresis loss values at each sample’s *E*_b_ field.

### Phase-field simulations

In the 3D phase-field modeling of the PZO system, the three components of the polarization serve as order parameters. The temporal evolution of polarization vector *P_i_* is governed by the time-dependent Ginzburg-Landau equation (*38*)

$$\frac{\partial P_{i}\left(r,t\right)}{\partial t}=-L_{i}\frac{\delta F}{\delta P_{i}\left(r,t\right)}+\xi_{i}\left(r,t\right),\left(i=1,2,3\right)$$ (4)

where *L* assumes the role of the kinetic coefficient associated with the domain wall mobility and $\delta F/\delta P_{i}\left(r,t\right)$ signifies the driving force for the spatial and temporal evolution of *P_i_*. $\xi_{i}\left(r,t\right)$ represents the Gaussian random fluctuation, satisfying the conditions $\left\langle \xi_{i}\left(r,t\right)\right\rangle =0$ and $\left\langle \xi_{i}\left(r,t\right)\xi_{j}\left(r^{\prime },t^{\prime }\right)\right\rangle =2k_{B}TL_{i}\delta_{\mathrm{ij}}\delta \left(r-r^{\prime }\right)\delta \left(t-t^{\prime }\right)$, where *k_B_* is the Boltzmann constant. The total free energy *F* encompasses contributions from bulk, gradient, elastic, and electrostatic energies, formulated as

$$F=∭\left(f_{\text{bulk}}+f_{\text{grad}}+f_{\text{elas}}+f_{\text{elec}}\right)\mathrm{dV}$$ (5)

where *V* is the volume of the film. The bulk free energy density is expressed as $f_{\text{bulk}}=\alpha_{\mathrm{ij}}P_{i}P_{j}+\alpha_{\text{ijkl}}P_{i}P_{j}P_{k}P_{l}+\alpha_{\text{ijklmn}}P_{i}P_{j}P_{k}P_{l}P_{m}P_{n}$, where α*_i_*, α*_ij_*, and α*_ijk_* are Landau coefficients. The expression for the gradient energy is given as $f_{\text{grad}}=-\gamma_{11}\theta_{i}^{2}\sum_{i}P_{i,i}^{2}-\gamma_{12}\theta_{i}^{2}\sum_{i\neq j\neq k}\left(P_{j,k}^{2}+P_{k,j}^{2}\right)+g_{11}\sum_{i}{\left(\frac{\partial^{2}P_{i}}{\partial x_{i}^{2}}\right)}^{2}+g_{12}\sum_{i\neq j}{\left(\frac{\partial^{2}P_{i}}{\partial x_{j}^{2}}\right)}^{2}$. The first two terms, describing the coupling between the oxygen tilt (θ) and polarization, characterize the tendency of neighboring polarizations to align antiparallel. The oxygen tilt is estimated as $\theta =\sqrt{-\frac{k_{0}}{b_{0}}\left(T-T_{\theta }\right)}$, where *T*_θ_ is the transition temperature of the oxygen tilt. $k_{0}$ and $b_{0}$ are positive material constants representing the coefficients of the quadratic and quartic terms in the potential energy expression for the oxygen tilt $F_{\theta }=\sum_{i}\left(\frac{k_{0}\left(T-T_{\theta }\right)}{2}\theta_{i}^{2}+\frac{b_{0}}{4}\theta_{i}^{4}\right)$. The last two terms, accounting for next-nearest neighbor interactions, play a critical role in driving the AFE-FE phase transition (*39*, *40*). The elastic energy density can be written as $f_{\text{elas}}=\frac{1}{2}C_{\text{ijkl}}\left(\varepsilon_{\mathrm{ij}}-\varepsilon_{\mathrm{ij}}^{0}\right)\left(\varepsilon_{\mathrm{kl}}-\varepsilon_{\mathrm{kl}}^{0}\right)$, in which ε*_ij_* is the total strain and $\varepsilon_{\mathrm{ij}}^{0}$ is the spontaneous strain. The spontaneous strain is related to be the polarization through electrostrictive coefficients *Q_ijkl_*: $\varepsilon_{\mathrm{ij}}^{0}=Q_{\text{ijkl}}P_{k}P_{l}$. The last term is the electrostatic energy density $f_{\text{elec}}=-\frac{1}{2}\varepsilon_{0}\varepsilon_{b}E_{i}^{2}-E_{i}P_{i}$, where ε_0_ is the permittivity of vacuum and ε*_b_* is the background dielectric constant (*41*).

The material parameters of PZO are sourced from prior literatures (*39*, *40*): The Landau parameters are given by $\alpha_{1}=2.61\times 10^{5}\left(T-T_{0}\right)$, where *T*_0_ is the Curie temperature (510 K), $a_{11}=5.60\times 10^{5}\;J\;m^{5}\;C^{-4}$, $a_{12}=2.89\times 10^{8}\;J\;m^{5}\;C^{-4}$, $a_{111}=1.65\times 10^{9}\;J\;m^{9}\;C^{-6}$, $a_{112}=-8.66\times 10^{8}\;J\;m^{9}\;C^{-6}$, and $a_{123}=3.19\times 10^{8}\;J\;m^{9}\;C^{-6}$. The elastic constants are $C_{11}=15.6\times 10^{10}\;N\;m^{-2}$, $C_{12}=9.6\times 10^{10}\;N\;m^{-2}$, and $C_{44}=12.7\times 10^{10}\;N\;m^{-2}$. The electrostrictive coefficients are $Q_{11}=0.048\;m^{4}\;C^{-2}$, $Q_{12}=0.015\;m^{4}\;C^{-2}$, $Q_{44}=0.047\;m^{4}\;C^{-2}$. To effectively capture the experimentally observed temperature-dependent tetragonality of AFE PZO, we linearly fitted the temperature dependent coefficients of the first-order gradient energy $\gamma_{11}\theta_{i}^{2}$ and $\gamma_{12}\theta_{i}^{2}$, yielding $\gamma_{11}\theta_{i}^{2}=\gamma_{12}\theta_{i}^{2}=12.94a_{c}^{4}\times 10^{7}\;J\;m\;C^{-2}$ at 300 K and $\gamma_{11}\theta_{i}^{2}=\gamma_{12}\theta_{i}^{2}=8.65a_{c}^{4}\times 10^{7}\;J\;m\;C^{-2}$ at 500 K, while the second-order gradient coefficient was fixed at $g_{11}=g_{12}=3.32a_{c}^{4}\times 10^{7}\;J\;m\;C^{-2}$. *a_c_* is the size of the mesh grid, 0.416 nm.

We implemented the phase-field equations via the finite element method using the open-source FERRET package (*42*) built on the multiphysics object-oriented simulation environment framework (*43*). The simulation size is 64Δx×64Δx×2Δx with a finite element mesh spacing of ∆x=0.0146 nm, corresponding to the cubic lattice constant of PZO. We applied periodic boundary conditions in-plane and imposed stress-free conditions on the displacement field out-of-plane while fixing the electrostatic potential at Φ=0. For the coupled variable system, we used third-order Hermite finite elements. We solved the resulting nonlinear algebraic equations with the Newton-Raphson method, using the generalized minimal residual method to handle the linear systems. We address time-dependent evolution using the implicit Euler method. The initial condition for all simulations was a paraelectric state with a small, randomly distributed noise.

### First-principles calculations

We performed first-principles calculations within the framework of the density functional theory, as implemented in the Vienna Ab initio Simulation Package code (*44*). We described interactions between ions and valence electrons using the projector-augmented wave formalism (*45*) and treated the exchange-correlation functional using the generalized gradient approximation in the Perdew-Burke-Ernzerhof (PBE) formulation (*46*). We used a 540-eV energy cutoff for the plane-wave basis and explicitly treated Pb(6*s*6*p*), Zr(4*s*4*p*4*d*5*s*), and O(2*s*2*p*) as valence states. For the AFE phase (40 atoms, Pbam space group), we used a 6 × 3 × 4 k-point mesh. To model Pb volatilization, we removed one Pb atom from eight-unit-cell superstructure. We fully relaxed all crystal structures with convergence thresholds of 10^−6^ eV for energy and 0.01 eV/Å for forces. While PBE underestimates bandgaps, comparative analysis of different configurations remains valid.

## Supplementary Materials

### This PDF file includes:

Figs. S1 to S24 Tables S1 and S2 References
