# Supplementary material for: Flash annealing–engineered wafer-scale relaxor antiferroelectrics for enhanced energy storage performance
Source: Sci Adv. 2025 Nov 14;11(46):eady2349. doi: 10.1126/sciadv.ady2349 (PMC12617463; doi:10.1126/sciadv.ady2349)
Supplement: Supplementary file 1 — Figs. S1 to S24 Tables S1 and S2 References [file sciadv.ady2349_sm.pdf]

Science Advances
AAAS

Supplementary Materials for

Flash annealing–engineered wafer-scale relaxor antiferroelectrics for enhanced energy storage performance

Yizhuo Li *et al.*

Corresponding author: Weijin Hu, wjhu@imr.ac.cn

Sci. Adv. **11**, eady2349 (2025)
DOI: 10.1126/sciadv.ady2349

This PDF file includes:

Figs. S1 to S24
Tables S1 and S2
References

Supplementary Materials

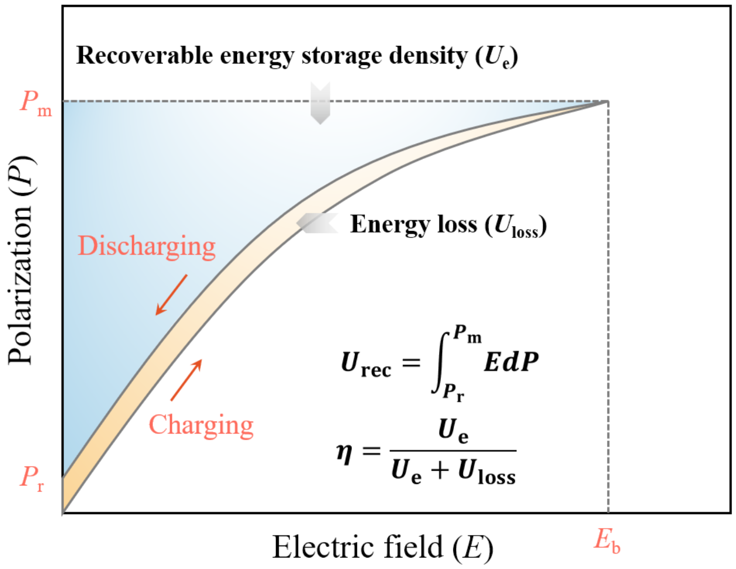

Fig. S1. Working principle of energy storage capacitor. The dischargeable energy density U_e is depicted by the olive area, which is affected by both the polarization P and the breakdown strength E_b of the dielectric, as expressed by the equation where P_m and P_r indicate the maximum and remnant polarization, respectively. The pink area symbolizes the energy loss U_{loss} from the hysteretic polarization switching during the charging/discharging cycle, while the energy storage efficiency η is given by U_e / (U_e + U_{loss}).

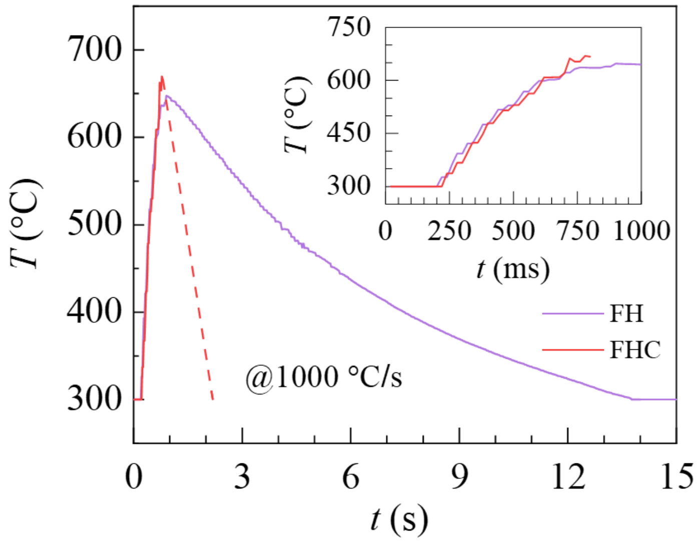

Fig. S2. The sample temperature monitored by an infrared thermometer for FH and FHC process. The inset provides a detailed view of the temperature rise during the heating process. Note 300 ºC is the lower detection limit of our infrared thermometer. Utilizing the Flash Heating and Cooling (FHC) and Flash Heating (FH) techniques, the heating rate can achieve an impressive 1000 ℃/s. It requires merely 650 milliseconds to reach the desired target temperature of 650 ℃. Moreover, by employing liquid nitrogen (LN2) quenching, FHC process achieves nearly instantaneous cooling within 1 second. While the FH treatment cool the sample in air with a cooling rate of ~30 ºC/s, requiring a cooling time of ~13 s.

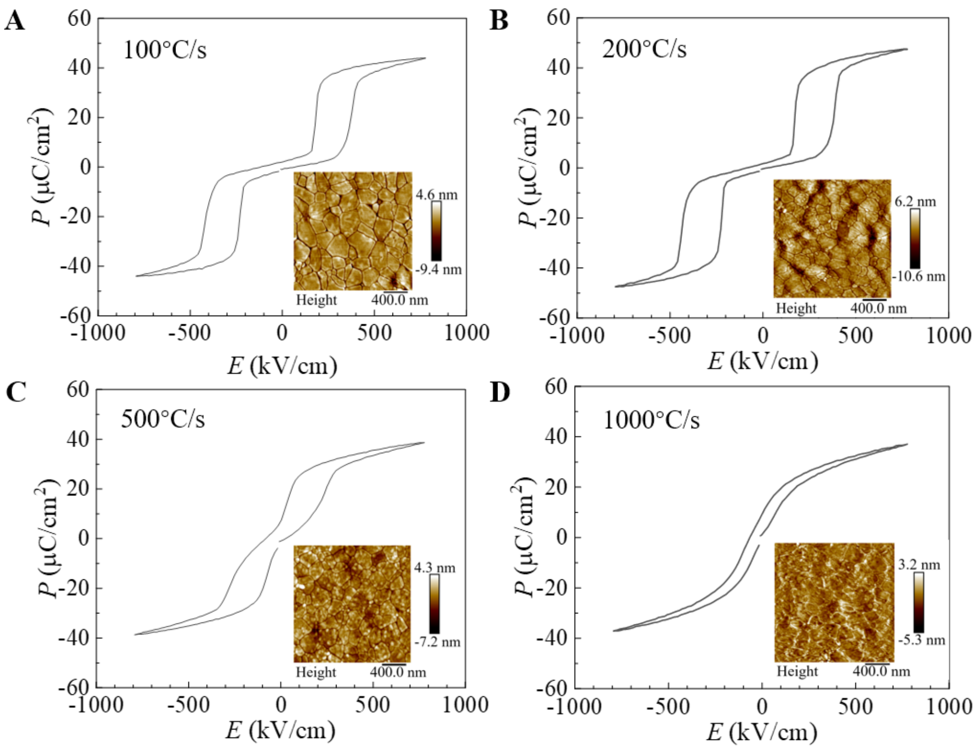

Fig. S3. *P-E* loops with various heating rates used in FHC process. (**A**) 100 ℃/s, (**B**) 200 ℃/s, (**C**) 500 ℃/s and (**D**) 1000 ℃/s. The insets present the AFM surface images of the corresponding films. Attaining a relaxor antiferroelectric state is possible via FHC treatment, however, this is conditional upon reaching an ultra-high heating rate of 1000 °C/s. Concurrently, the densification of grain boundaries experiences a marked improvement with increasing the heating rate.

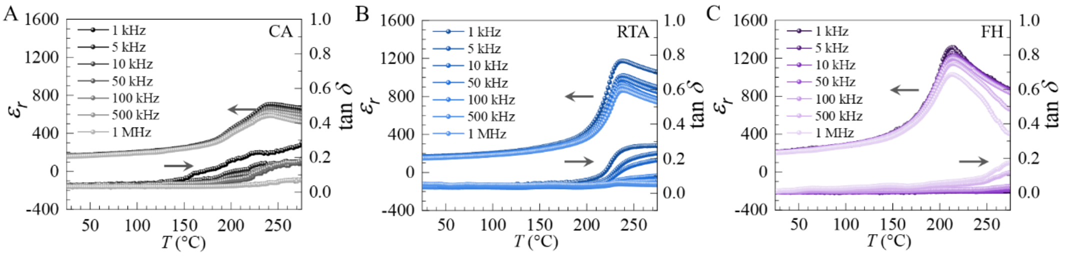

Fig. S4. The temperature dependent dielectric spectra of PZO films treated by various processes. A. CA. B. RTA. C. FH. Left, the dielectric constant (ε_r); and right, the dielectric loss (tan δ). We applied an 1 V ac voltage during the measurement. The frequency ranges from 1 kHz to 1 MHz. With increasing the heating rate from CA, RTA to FH, distinct dialectic constant enhancement has been observed, particularly near the Curie temperature region.

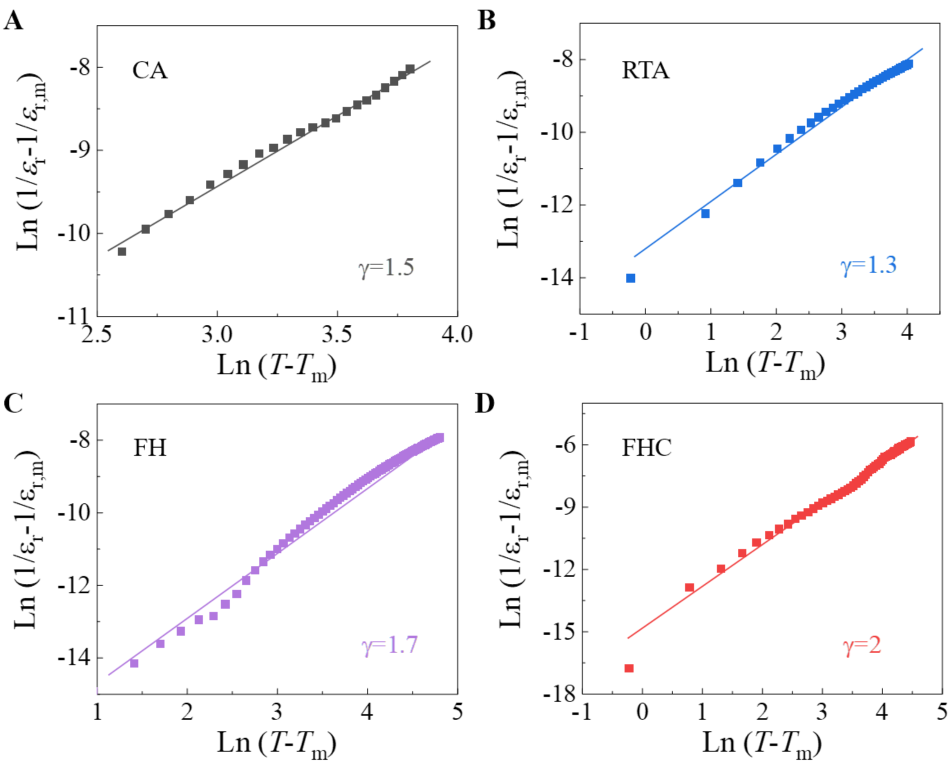

Fig. S5. The relaxor diffuseness factor γ determined from temperature-dependent dielectric data at 1 kHz. (**A**) CA, (**B**) RTA, (**C**) FH and (**D**) FHC. The solid lines are the fittings according to the modified Curie-Weiss law, $1/\varepsilon_r - 1/\varepsilon_{r,\,m} = (T - T_m)^{\gamma}/C$, where ε_r is the permittivity at temperature T, T_m is the temperature at which the permittivity reaches its maximum value $\varepsilon_{r,\,m}$, C is a constant, and γ is the relaxor diffuseness factor. γ ranges from 1 for normal antiferroelectric to 2 for an ideal relaxor antiferroelectric. This analytical approach provides a quantitative assessment of relaxor behavior, offering valuable insights for understanding the properties of PZO films.

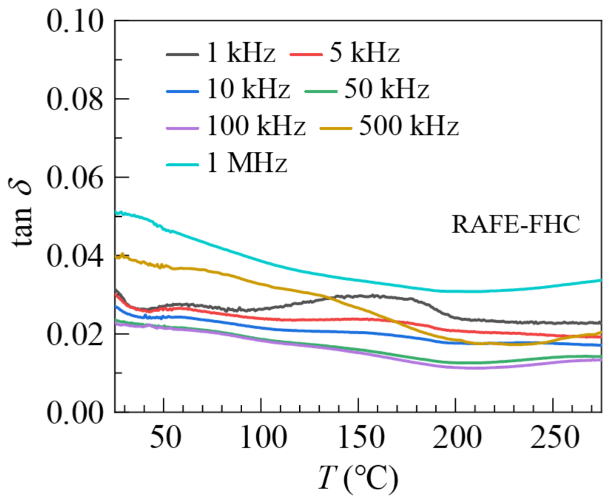

Fig. S6. The enlarged dielectric loss as a function of temperature for PZO film treated by FHC process. FHC-film shows minor frequency dispersion in dielectric loss. There are several reasons. First, abundant sub-grain boundaries and grain boundaries likely pin domain walls, impeding dipole rotation under ac electric fields. Second, the present FHC-film has a crystal structure closing to that of cubic paraelectric phase, suppressing the frequency dependence.

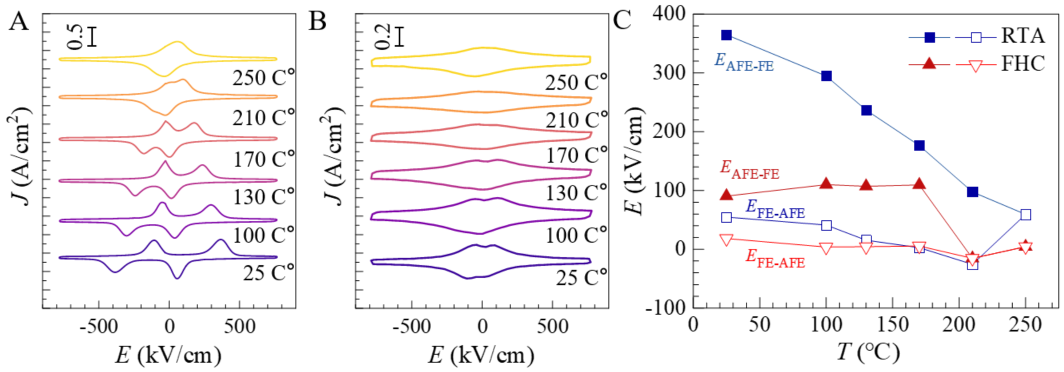

Fig. S7. The temperature dependent AFE-FE transition behavior of PZO films. Current switching curves at different temperatures for (**A**) RTA film, and (**B**) FHC film. (**C**) The AFE-FE transition field ($E_{\text{AFE-FE}}$) and FE-AFE back-transition field ($E_{\text{FE-AFE}}$) as a function of temperature for RTA and FHC film. The transition fields are determined from the peak positions of the switching current presented in **A** and **B**. Four distinct current peaks for RTA film at room temperature corelates to the sharp AFE-FE transition and FE-AFE transition in the double-hysteresis loop (**Fig. S7A**). These peaks gradually merge together with increasing the temperature above T_c of ~ 238 °C. In contrast, though four current switching peaks exist for FHC film, their current densities are relative weak, and their peak positions are close to 0 kV/cm, due to the relaxer feature of FHC film (**Fig. S7B**). Therefore, FHC-film possesses smaller transition fields ($E_{\text{AFE-FE}}$ and $E_{\text{FE-AFE}}$) that are almost independent on the temperature, confirming the relaxor behavior of FHC-film and the similarity between RAFE and PE state.

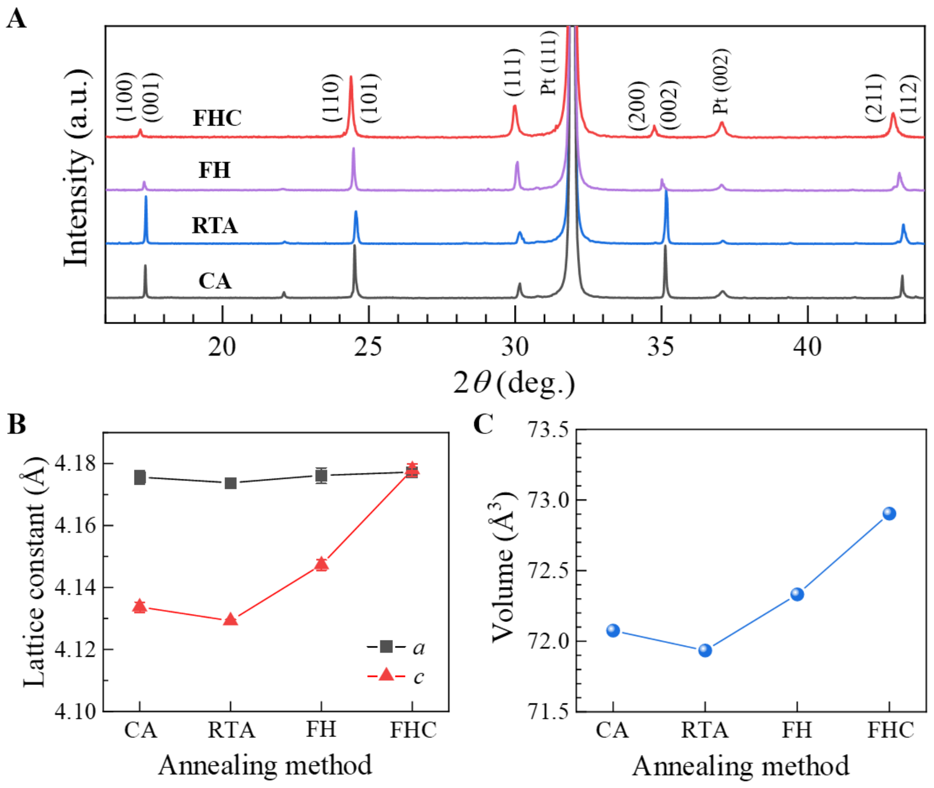

Fig. S8. Synchrotron HRXRD analysis. (**A**) HRXRD at room temperature by using an X-ray source with an energy of 10 keV. (**B** and **C**) The lattice constants (**B**) and the unit-cell volume (**C**) derived from (**A**). All films exhibit a polycrystalline nature with diffraction patterns arising from various crystal planes. As the heating rate is increased from conventional annealing (CA), rapid thermal annealing (RTA), to flash heating (FH) and flash heating and cooling (FHC), the diffraction peaks shift to lower angles. This shift corresponds to a volume expansion from ~ 72.1 $Å^3$ to 73 $Å^3$, representing an increase of 1.25%. This volume expansion is predominantly due to the expansion of the c lattice parameter from ~ 4.135 Å to 4.180 Å, which is a 1.1% increase. The lattice constants a and c are nearly equivalent in the FHC film, suggesting a strong resemblance to the high-temperature paraelectric phase. This demonstrates that FHC process can effectively preserve the high-temperature crystal structure down to room temperature.

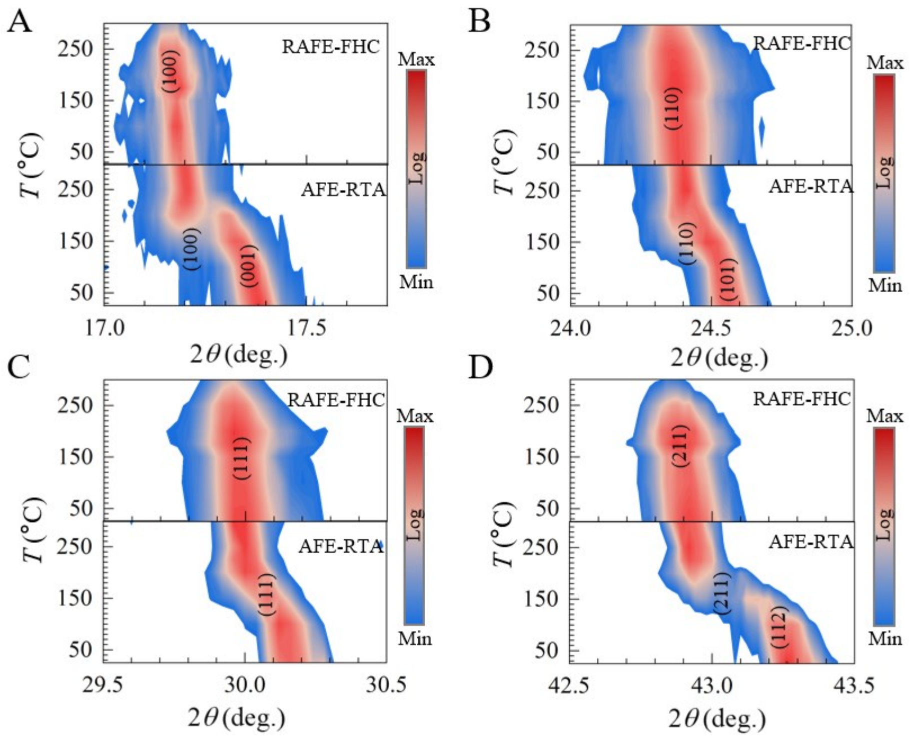

Fig. S9. The temperature dependent diffraction peaks for various crystal planes. A. (100)//(001). B. (110)//(101). C. (111) . D. (211)//(112). Distinct peak splitting is observed for (001) and (112) diffraction planes for RTA film, suggesting the tetragonal distortion because of the inequivalence of lattice *a* (or *b*) and *c*. The relative strong intensity of (001) over that of (100) indicate the preferred orientation exists in the film.

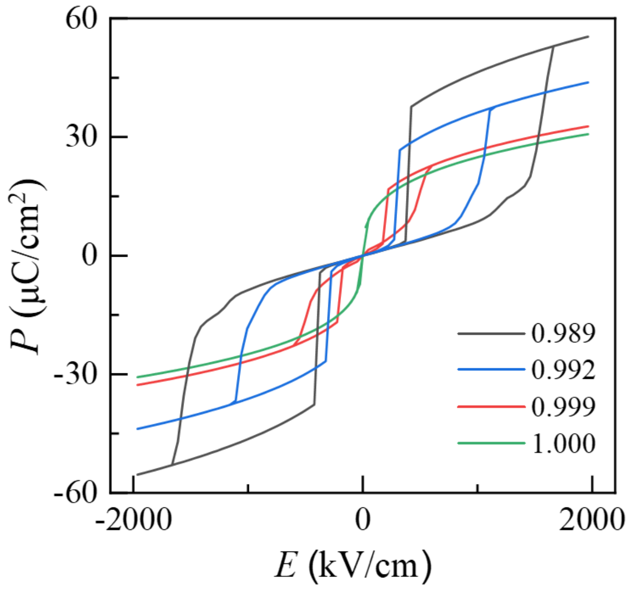

Fig. S10. *P-E* loops at various c/a ratios predicted by phase-field simulation. As the c/a ratio increases, the characteristic double-hysteresis *P-E* loops indicative of the antiferroelectric phase transits into the narrower, characteristic relaxor loops for the relaxor antiferroelectric phase.

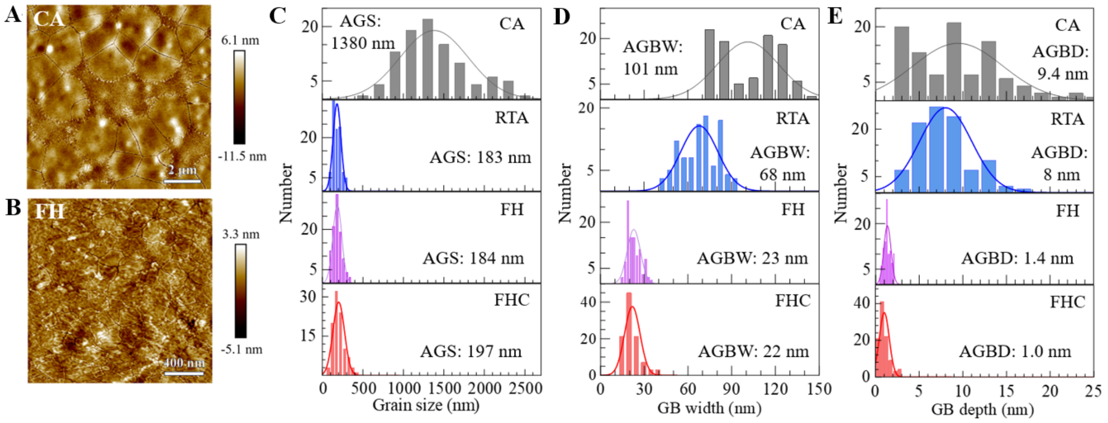

Fig. S11. The grains and grain boundaries characteristics. (**A** and **B**) The surface images of PZO films treated by CA (**A**) and FH (**B**). (**C** to **E**) Distribution of grain size (**C**), grain boundary width (**D**), and grain boundary depth (**E**) for PZO films treated by various processes.

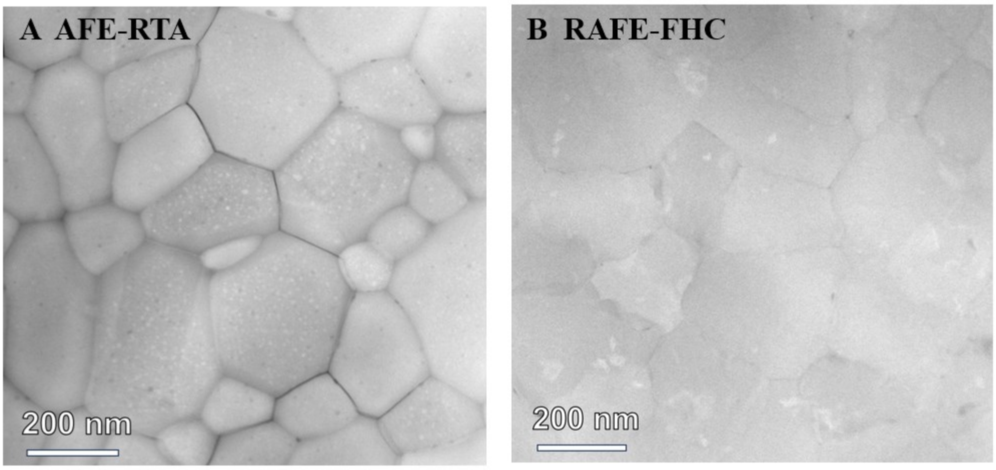

Fig. S12. STEM morphology images of (A) AFE-RTA film and (B) RAFE-FHC film. The AFE-RTA film exhibits polycrystalline characteristics, featuring regularly shaped and sharp-edged grains that are distinctly separated by grain boundaries. Conversely, the RAFE-FHC film, while also polycrystalline, presents a very smooth surface texture, with the grains not easily discernible due to the shallow and narrow grain boundaries. This results in an exceptionally compact and uniform surface.

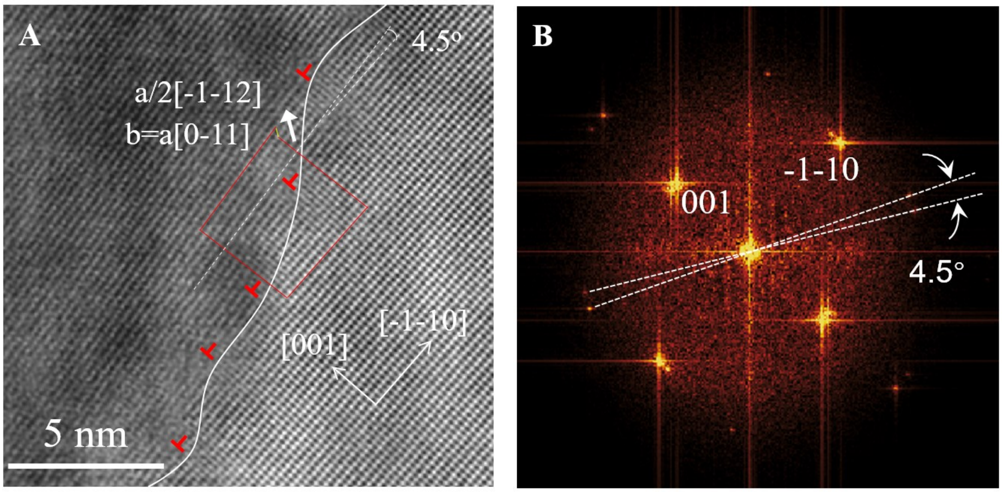

Fig. S13. HRTEM image of a low-angle GB in RAFE-FHC film. (**A**) The HAADF-STEM image. The solid line marks the GB position. (**B**) The diffraction pattern derived from (**A**) via Fast Fourier transformation. Two distinct sets of diffraction spots are evident in the fast FFT pattern, enabling us to determine the misorientation angle of this GB to be 4.5°. Following the grain boundary as indicated by the white line in (**A**), we have identified a series of perfect dislocations with a Burgers vector of a [0-11]. This finding indicates that the formation of GB is a consequence of dislocation pile-up at the boundary.

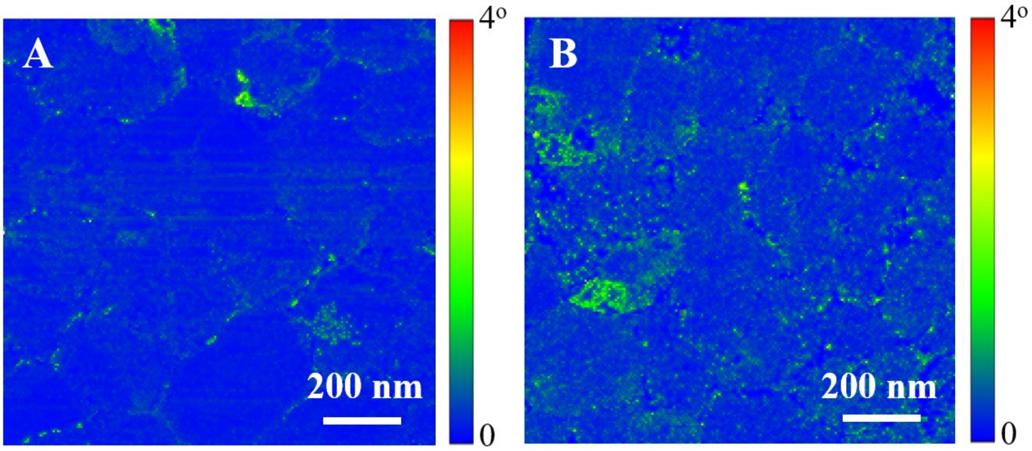

Fig. S14. Kernel Average Misorientation (KAM) mappings for PZO films treated by (A) RTA process, and (B) FHC process. Compared to RTA-film, larger intra-grain misorientation angles appear in FHC-film, particularly at grain boundaries and within sub-grains, suggesting higher residual stress levels in FHC-film.

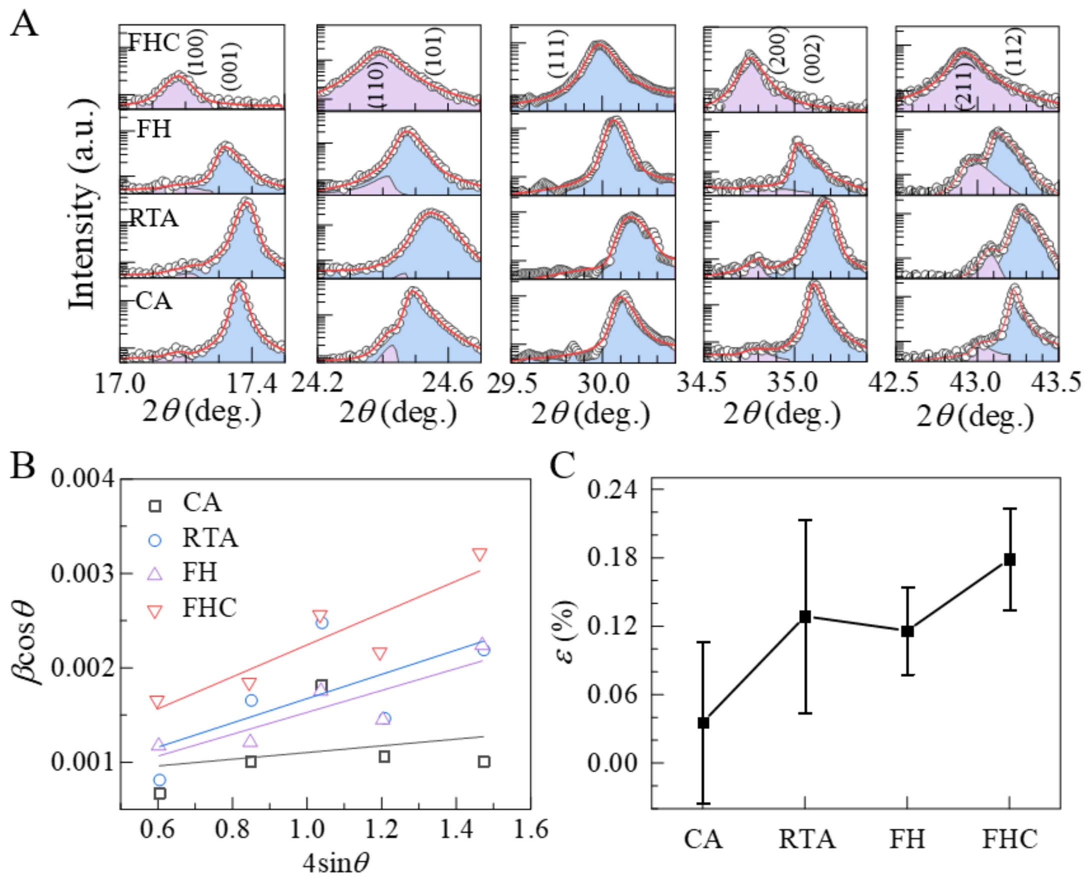

Fig. S15. X-ray diffraction broadening analysis. A. The enlarged XRD peaks at (100)/(001), (110)/(101), (111), (200)/(002), and (211)/(112) for PZO films treated by CA, RTA, FH, and FHC processes. B. Williamson-Hall plot (βcos θ *vs.* 4sin θ) for all samples with β derived from A. C. Microscopic strain ϵ derived from the slope of the curves shown in B.

To quantify the internal stress, we analyzed the peak broadening (characterized by Full Width at Half Maximum, FWHM) of room-temperature XRD patterns across different films (**Fig. S8A**). This is based on the fact that strain-induced lattice distortions would widen the diffraction peaks. However, we mention that this broadening could also arise from finite crystallite size (Scherrer effect) and instrumental broadening. Here we use Williamson-Hall (W-H) method to decouple these contributions, which states that the total broadening of the XRD peak follows equation $\beta \cos\theta = \frac{K\lambda}{D} + 4\varepsilon \sin\theta$, with the first term represents the peak broadening induced by the crystallite size and the second term by the strain. β is the FWHM of the diffraction peak, θ is the diffraction peak, K is the Scherrer factor, λ is the wavelength, and ϵ is the strain. As evidenced in **Fig. S15A**, the enlarged diffraction peaks of PZO films [(100)/(001), (110)/(101), (200)/(002), and (211)/(112)] reveal distinct peak splitting for CA-, RTA-, and FH-processed films-a characteristic signature of tetragonal distortion. Whereas, FHC film shows minor peak splitting. By fitting these diffraction peaks with Pseudo-Voigt function, we obtain β values and plot β cos θ *vs.* 4 sin θ as shown in **Fig. S15B**, from which we derive the microscopic strain ϵ from the slope of

each curve and summarize them in **Fig. S15C**. ϵ increases from 0.036% for CA, 0.13% for RTA, 0.12% for FH, and ultimately to 0.18% for FHC, quantitatively demonstrating the critical role of heating/cooling rate in tailoring the microstructure of PZO films. As evidenced by our analysis, the flash heating/cooling during FHC process generates a large amount of sub-grain boundaries (**Fig. 3F**) together with dislocation networks (**Fig. 3G & Fig. S13**), resulting in more statistical fluctuations in lattice constants, manifesting as enhanced microscopic stress that stabilize the high-temperature cubic-like phase.

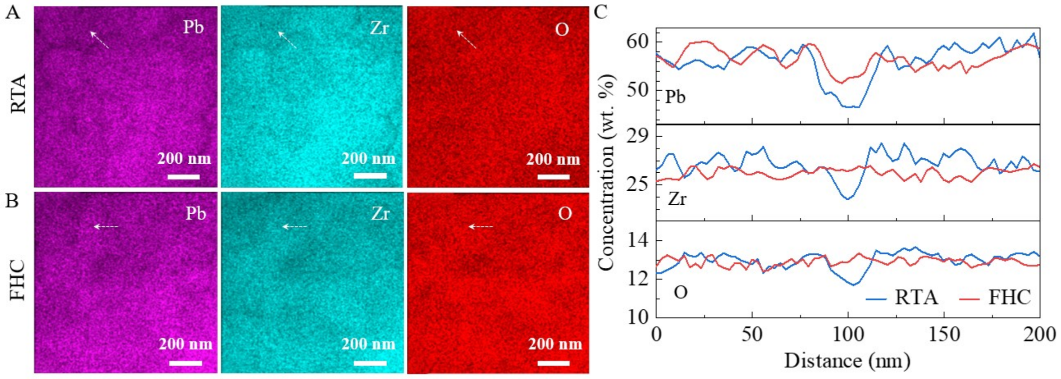

Fig. S16. EDS mapping of PZO films. (A) RTA film and (B) FHC film. (C) The element line scans along the typical grain boundaries (indicated by dashed arrows in A and B) for RTA and FHC films. While both films show homogeneous element distributions within the grains, FHC-film shows minor Pb reduction on the grain boundaries as indicated by the minor intensity fluctuations across the grain boundaries, compared with that of RTA-film. This has also been confirmed from the line scan across the grain boundaries, as displayed in **Fig. S16C**.

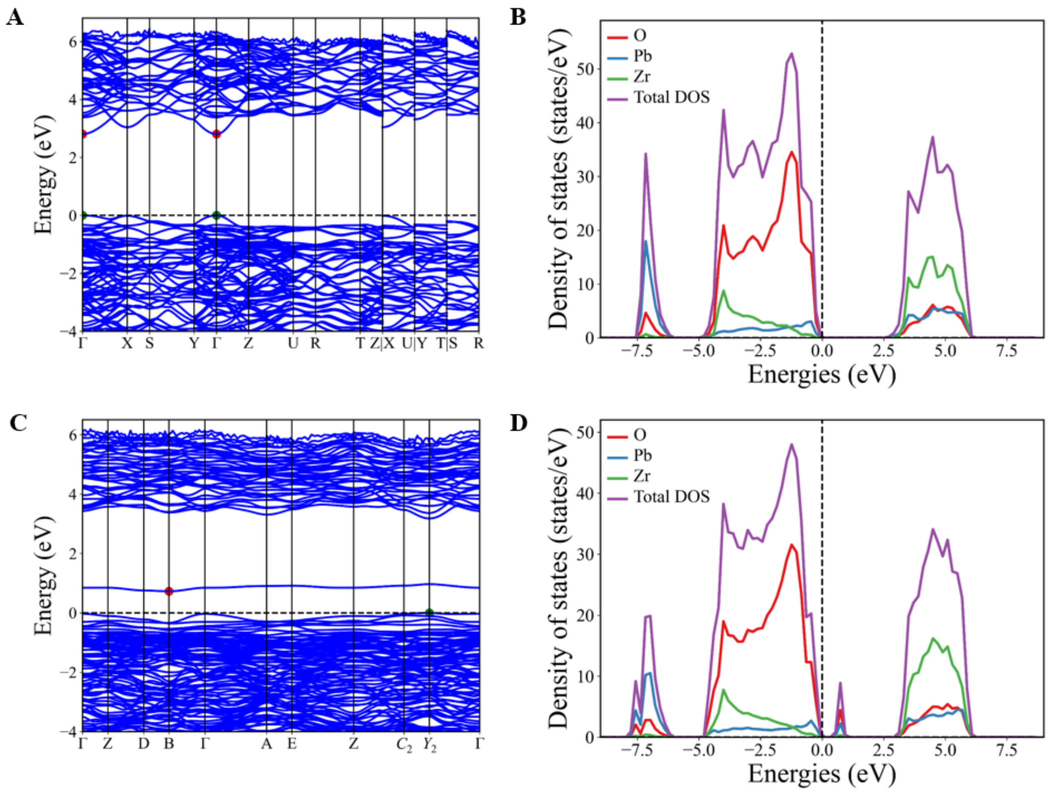

Fig. S17. Band structures and defects. (**A**) Band structure and (**B**) Partial density of states (DOS) of PZO film without considering point defects. (**C**) Band structure and (**D**) Partial DOS of PZO with Pb vacancies.

The calculated band structure and DOS in (**C**) and (**D**) confirms that Pb vacancy introduces an acceptor level at ~0.7 eV above the valence band maximum (VBM). The 0.2 eV discrepancy between this theoretical value and experimental observations can be attributed to the limitations of the PBE functional method, which does not adequately account for strong-correlation electronic systems. The observed O orbital contribution in the in-gap states is attributed to local structural and electronic perturbations around the Pb vacancy, and such coupling between Pb and O is consistent with defect configurations reported in perovskite systems, where Pb-O defect complexes are known to form under Pb-deficient conditions (*47, 48*).

The bandgap of the RAFE film is approximately 3.06 eV, which is ~0.3 eV smaller than that of the normal AFE-PZO film. This reduction is ascribed to the combined effect of lattice constants and Pb vacancy concentrations, as supported by the bandgap values summarized in **Table S2**.

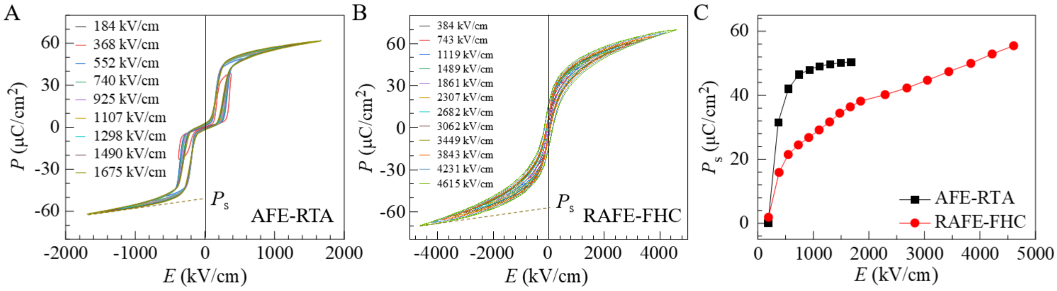

Fig. S18. *P-E* loops under various maximum electric fields. A. for AFE-RTA film, B. for RAFE-FHC film, and C. the saturation polarization P_s as a function of electric field. Here P_s is determined by extrapolating the high-field linear part to zero field as depicted by dashed lines in A and B. For small electric field of ~200 kV/cm, P_s equals to 0 both for RTA and FHC films because they are in the AFE or RAFE ground state. Increasing the electric field will see the sharp increase of P_s for RTA film because of the sharp field-induced AFE to FE transition. In contrast, the increase of P_s in FHC film is slowly because of its relaxor feature. However, P_s of FHC film will finally exceeds that of RTA film when increasing the electric field further, because it can sustain larger electric field without breaking.

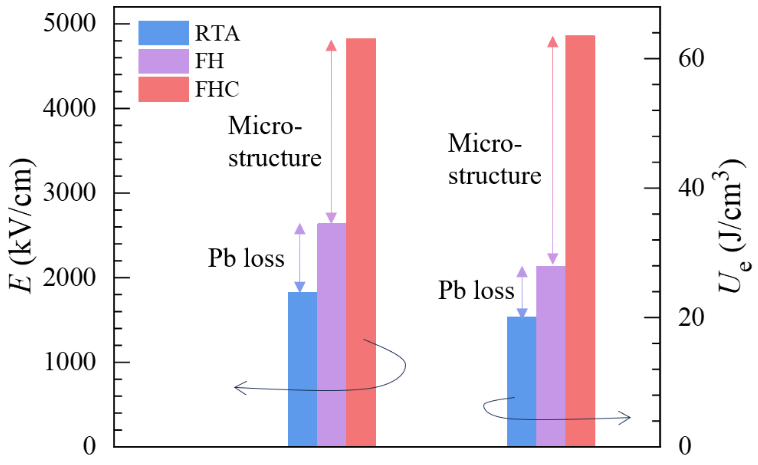

Fig. S19. Role of Pb deficiency vs. microstructure in energy storage performance. Breakdown strength (E_b, left) and energy storage density (U_e, right). Microstructural factors include sub-grains and nanodomains. **Fig. 3H** shows the Pb/Zr ratios of various films processed by different methods. Serious Pb deficiency occurs in RTA-treated films, while FH- and FHC-processed films maintain stoichiometric Pb. Notably, RTA- and FH-treated films exhibit AFE characteristics, whereas FHC yields a RAFE state due to its sub-grain microstructure and associated nano-domains. Therefore, by comparing RTA- and FH-treated films, we can isolate the impact of Pb deficiency on the device performance; while by comparing FH- and FHC-treated films, we can estimate the contribution from changes in microstructure and domain scale. **Fig. S19** quantifies these effects through breakdown electric field E_b (left) and energy storage density U_e (right). Eliminating Pb loss enhances both E_b and U_e, but microstructure optimization drives substantially greater improvement. We therefore conclude that microstructural engineering dominates energy storage enhancement.

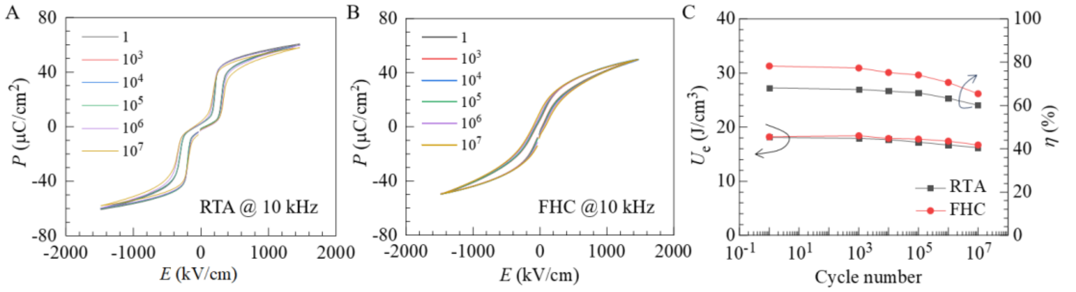

Fig. S20. Fatigue performance of the PZO capacitors. *P-E* loops at 10 kHz and room temperature during the fatigue measurement for (A) AFE-RTA film and (B) RAFE-FHC film. We employed a triangle waveform electric field of 1500 kV/cm at 500 kHz to ensure full polarization switching. C. The energy storage density (U_e, left) and efficiency (η, right) as a function of cycling numbers derived from (A) and (B). As shown in (A) and (B), both RTA and FHC devices exhibit minimal degradation in their *P-E* loops after 10^7 cycles, demonstrating excellent energy storage stability (C). Specifically, the energy storage density (U_e) degraded by 10.5% (RTA) and 8.2% (FHC), and the efficiency (η) degraded by 11.7% (RTA) and 16.4% (FHC) after 10^7 endurance cycles.

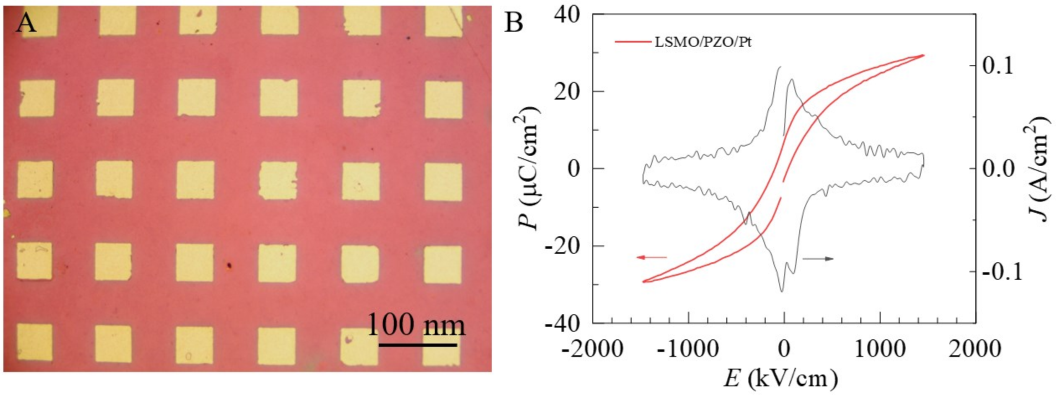

Fig. S21. Ultrafast crystallization of the LSMO/PZO-300 nm/Pt capacitor device. (A) Optical image of the devices after FHC process. (B) *P*-*E* hysteresis loop (left) and the corresponding switching current loop (right) of a representative device. Here we treat the entire capacitor devices (not solely the PZO film) directly by the FHC process. As shown in the optical image (A), the devices remain in good condition following FHC, and they exhibit relaxor antiferroelectric hysteresis loops as expected.

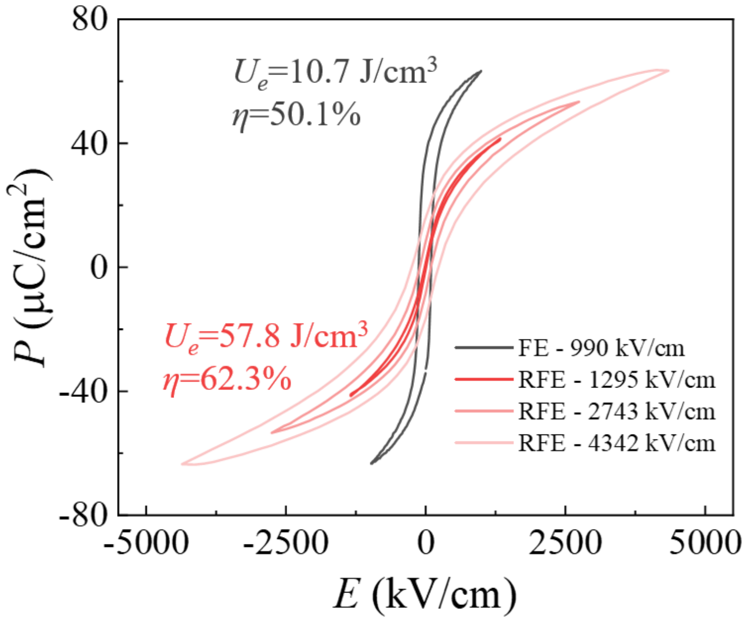

Fig. S22. *P-E* loops of PZT films treated by RTA and FHC. Utilizing the FHC method, we can transform the PZT film from the ferroelectric phase (FE) to the relaxor ferroelectric (RFE) phase. In conjunction with this phase transition, there is a marked enhancement in energy storage performance, with U_e and η increasing from 10.7 J/cm^3 and 50.1% for the ferroelectric phase to 57.8 J/cm^3 and 62.3% for the relaxor ferroelectric phase, respectively.

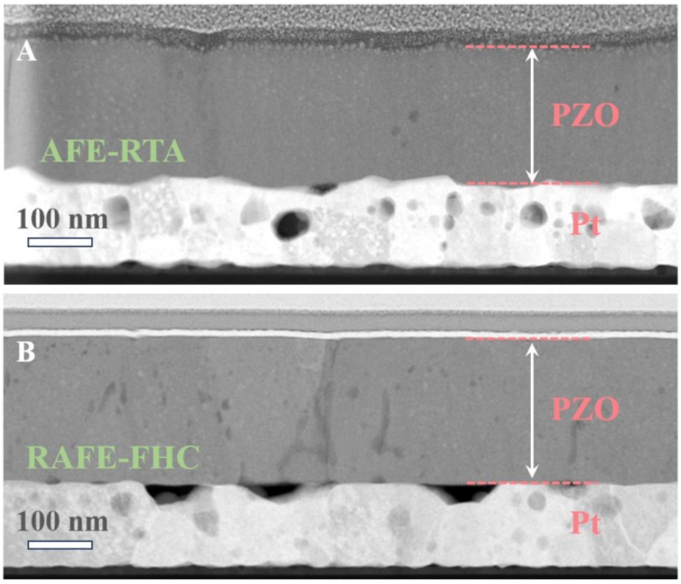

Fig. S23. TEM cross-sectional images. (**A**) AFE-RTA and (**B**) RAFE-FHC films. The films fabricated by both methods exhibit the same thickness of ~260 nm.

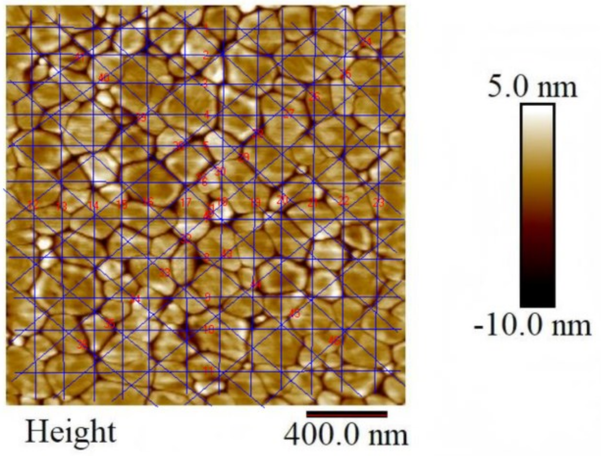

Fig. S24. Method for grain size statistical analysis. We analyzed grain size from AFM surface image using the intercept method (Heyn method) implemented in Nano Measurer software. This approach determines the average grain size ($\bar{d}$) by calculating the ratio of the total length of random test lines (L) to the number of grain boundary intersections (N), expressed as $\bar{d} = L/N$. The derived mean intercept length corresponds to the effective grain diameter. For anisotropic microstructures, measurements included both horizontal and vertical test line orientations (as shown by blue lines in AFM image) to obtain a weighted average that accounts for grain morphology. The analysis yielded grain size distribution histograms and statistical parameters, providing quantitative characterization of both equiaxed and non-equiaxed grain structure.

Table S1. Typical parameters used in various processing conditions.

Processing method	Heating rate (℃/s)	Heating time (s)	Temp. (℃)	Holding time (s)	Cooling method	Cooling time (s)
CA	1	700	700	1800	air	1000
RTA	30	22	650	180	air	1000
FH	1000	0.65	650	0	air	20
FHC	1000	0.65	650	0	liquid nitrogen	<1

Table S2. Summary of bandgap (E_g) values from first-principles calculations and experimental measurements. For first-principles calculations, we use the Perdew-Burke-Ernzerhof (PBE) functional method, and consider the effect of lattice parameters and Pb vacancies (V_{Pb}). The lattice parameters are taken from experimental results. It is found that RTA film with a V_{Pb} concentration of 12.5% has a E_g of 3.20 eV, which is ~0.6 eV larger than that of FHC film (E_g ~ 2.61 eV). While experimentally, RTA film has a E_g of 3.37 eV, which is ~0.3 eV larger than that of FHC film (E_g ~ 3.06 eV). Therefore, PBE calculations successfully reproduce the experimental trend of bandgap reduction in FHC film relative to RTA film, despite systematically underestimating the bandgap values due to the known bandgap limitation of PBE functionals.

Film	*a* (Å)	*c* (Å)	V_{Pb}	E_g cal. (eV)	E_g exp. (eV)
AFE-RTA	4.17	4.13	No	2.73	
AFE-RTA	4.17	4.13	Yes, 12.5%	3.20	3.37
RAFE-FHC	4.18	4.18	No	2.61	3.06

REFERENCES AND NOTES

1. B. Chu, X. Zhou, K. Ren, B. Neese, M. Lin, Q. Wang, F. Bauer, Q. M. Zhang, A dielectric polymer with high electric energy density and fast discharge speed. *Science* **313**, 334–336 (2006).

2. H. Wu, F. Zhuo, H. Qiao, L. Kodumudi Venkataraman, M. Zheng, S. Wang, H. Huang, B. Li, X. Mao, Q. Zhang, Polymer-/ceramic-based dielectric composites for energy storage and conversion. *Energy Environ. Mater.* **5**, 486–514 (2022).

3. G. Wang, Z. Lu, Y. Li, L. Li, H. Ji, A. Feteira, D. Zhou, D. Wang, S. Zhang, I. M. Reaney, Electroceramics for high-energy density capacitors: Current status and future perspectives. *Chem. Rev.* **121**, 6124–6172 (2021).

4. P. Simon, Y. Gogotsi, Materials for electrochemical capacitors. *Nat. Mater.* **7**, 845–854 (2008).

5. H. Pan, S. Lan, S. Xu, Q. Zhang, H. Yao, Y. Liu, F. Meng, E.-J. Guo, L. Gu, D. Yi, X. R. Wang, H. Huang, J. L. Mac Manus-Driscoll, L.-Q. Chen, K.-J. Jin, C.-W. Nan, Y.-H. Lin, Ultrahigh energy storage in superparaelectric relaxor ferroelectrics. *Science* **374**, 100–104 (2021).

6. J. Li, Z. Shen, X. Chen, S. Yang, W. Zhou, M. Wang, L. Wang, Q. Kou, Y. Liu, Q. Li, Z. Xu, Y. Chang, S. Zhang, F. Li, Grain-orientation-engineered multilayer ceramic capacitors for energy storage applications. *Nat. Mater.* **19**, 999–1005 (2020).

7. B. Yang, Q. Zhang, H. Huang, H. Pan, W. Zhu, F. Meng, S. Lan, Y. Liu, B. Wei, Y. Liu, L. Yang, L. Gu, L. Q. Chen, C. W. Nan, Y. H. Lin, Engineering relaxors by entropy for high energy storage performance. *Nat. Energy* **8**, 956–964 (2023).

8. H. Palneedi, M. Peddigari, G.-T. Hwang, D.-Y. Jeong, J. Ryu, High-performance dielectric ceramic films for energy storage capacitors: Progress and outlook. *Adv. Funct. Mater.* **28**, 1803665 (2018).

9. A. R. Jayakrishnan, J. P. B. Silva, K. Kamakshi, D. Dastan, V. Annapureddy, M. Pereira, K. C. Sekhar, Are lead-free relaxor ferroelectric materials the most promising candidates for energy storage capacitors? *Prog. Mater Sci.* **132**, 101046 (2023).

10. B. Yang, Y. Liu, R. J. Jiang, S. Lan, S. Z. Liu, Z. Zhou, L. Dou, M. Zhang, H. Huang, L. Q. Chen, Y. L. Zhu, S. Zhang, X. L. Ma, C. W. Nan, Y. H. Lin, Enhanced energy storage in antiferroelectrics via antipolar frustration. *Nature* **637**, 1104–1110 (2025).

11. N. Luo, K. Han, M. J. Cabral, X. Liao, S. Zhang, C. Liao, G. Zhang, X. Chen, Q. Feng, J.-F. Li, Y. Wei, Constructing phase boundary in $AgNbO_3$ antiferroelectrics: Pathway simultaneously achieving high energy density and efficiency. *Nat. Commun.* **11**, 4824 (2020).

12. J. Kim, S. Saremi, M. Acharya, G. Velarde, E. Parsonnet, P. Donahue, A. Qualls, D. Garcia, L. W. Martin, Ultrahigh capacitive energy density in ion-bombarded relaxor ferroelectric films. *Science* **369**, 81–84 (2020).

13. T. Tunkasiri, G. Rujijanagul, Dielectric strength of fine grained barium titanate ceramics. *J. Mater. Sci. Lett.* **15**, 1767–1769 (1996).

14. L. Yang, X. Kong, F. Li, H. Hao, Z. Cheng, H. Liu, J.-F. Li, S. Zhang, Perovskite lead-free dielectrics for energy storage applications. *Prog. Mater Sci.* **102**, 72–108 (2019).

15. N. Luo, K. Han, F. Zhuo, C. Xu, G. Zhang, L. Liu, X. Chen, C. Hu, H. Zhou, Y. Wei, Aliovalent A-site engineered $AgNbO_3$ lead-free antiferroelectric ceramics toward superior energy storage density. *J. Mater. Chem. A* **7**, 14118–14128 (2019).

16. J. Ma, J. Zhang, J. Guo, X. Li, S. Guo, Y. Huan, J. Wang, S.-T. Zhang, Y. Wang, Achieving ultrahigh energy storage density in lead-free sodium niobate-based ceramics by modulating the antiferroelectric phase. *Chem. Mater.* **34**, 7313–7322 (2022).

17. B. Yang, Y. Zhang, H. Pan, W. Si, Q. Zhang, Z. Shen, Y. Yu, S. Lan, F. Meng, Y. Liu, H. Huang, J. He, L. Gu, S. Zhang, L. Q. Chen, J. Zhu, C. W. Nan, Y. H. Lin, High-entropy enhanced capacitive energy storage. *Nat. Mater.* **21**, 1074–1080 (2022).

18. F. Li, X. Hou, T. Li, R. Si, C. Wang, J. Zhai, Fine-grain induced outstanding energy storage performance in novel $Bi_{0.5}K_{0.5}TiO_3$–$Ba(Mg_{1/3}Nb_{2/3})O_3$ ceramics via a hot-pressing strategy. *J. Mater. Chem. C* **7**, 12127–12138 (2019).

19. L. Zhong, J. Wang, H. Sheng, Z. Zhang, S. X. Mao, Formation of monatomic metallic glasses through ultrafast liquid quenching. *Nature* **512**, 177–180 (2014).

20. H. T. Martirena, J. C. Burfoot, Grain-size and pressure effects on the dielectric and piezoelectric properties of hot-pressed PZT-5. *Ferroelectrics* **7**, 151–152 (1974).

21. T. Zhang, Z. Shi, C. Yin, C. Zhang, Y. Zhang, Y. Zhang, Q. Chen, Q. Chi, Tunable polarization-drived superior energy storage performance in $PbZrO_3$ thin films. *J. Adv. Ceram.* **12**, 930–942 (2023).

22. C. Yin, T. Zhang, Z. Shi, B. Zhang, C. Zhang, Q. Chi, Tunable polarization-drived high energy storage performances in flexible $PbZrO_3$ films by growing Al_2O_3 nanolayers. *J. Adv. Ceram.* **12**, 2123–2133 (2023).

23. E. Sawaguchi, Lattice constant of $PbZrO_3$. *J. Physical Soc. Japan* **7**, 110–111 (1952).

24. W. Bollmann, *Dislocation Networks—Subgrain Boundaries*. Springer (1970).

25. V. D. Mote, Y. Purushotham, B. N. Dole, Williamson-Hall analysis in estimation of lattice strain in nanometer-sized ZnO particles. *J. Theor. Appl. Phys.* **6**, 6 (2012).

26. J. W. McPherson, J. Kim, A. Shanware, H. Mogul, J. Rodriguez, Trends in the ultimate breakdown strength of high dielectric-constant materials. *IEEE Trans. Electron Devices* **50**, 1771–1778 (2003).

27. P. Jain, E. J. Rymaszewski, Embedded thin film capacitors-theoretical limits. *IEEE Trans. Adv. Packag.* **25**, 454–458 (2002).

28. J. Chen, Y. Zhou, X. Huang, C. Yu, D. Han, A. Wang, Y. Zhu, K. Shi, Q. Kang, P. Li, P. Jiang, X. Qian, H. Bao, S. Li, G. Wu, X. Zhu, Q. Wang, Ladderphane copolymers for high-temperature capacitive energy storage. *Nature* **615**, 62–66 (2023).

29. Q. Li, L. Chen, M. R. Gadinski, S. Zhang, G. Zhang, U. Li, E. Iagodkine, A. Haque, L. Q. Chen, N. Jackson, Q. Wang, Flexible high-temperature dielectric materials from polymer nanocomposites. *Nature* **523**, 576–579 (2015).

30. C. Yin, T. Zhang, B. Zhang, C. Zhang, Q. Chi, High energy storage performance for flexible $PbZrO_3$ thin films by seed layer engineering. *Ceram. Int.* **48**, 23840–23848 (2022).

31. J. Ge, D. Remiens, X. Dong, Y. Chen, J. Costecalde, F. Gao, F. Cao, G. Wang, Enhancement of energy storage in epitaxial $PbZrO_3$ antiferroelectric films using strain engineering. *Appl. Phys. Lett.* **105**, 112908 (2014).

32. D.-L. Ko, T. Hsin, Y.-H. Lai, S.-Z. Ho, Y. Zheng, R. Huang, H. Pan, Y.-C. Chen, Y.-H. Chu, High-stability transparent flexible energy storage based on $PbZrO_3$/muscovite heterostructure. *Nano Energy* **87**, 106149 (2021).

33. Y. Luo, C. Wang, C. Chen, Y. Gao, F. Sun, C. Li, X. Yin, C. Luo, U. Kentsch, X. Cai, M. Bai, Z. Fan, M. Qin, M. Zeng, J. Dai, G. Zhou, X. Lu, X. Lou, S. Zhou, X. Gao, D. Chen, J.-M. Liu, Tripling energy storage density through order–disorder transition induced polar nanoregions in $PbZrO_3$ thin films by ion implantation. *Appl. Phys. Rev.* **10**, 011403 (2023).

34. Y. Fang, Y. Bai, Y. Z. Li, N. Liu, F. Zhang, C. Wang, Z. J. Wang, Improved energy storage performance of $PbZrO_3$ antiferroelectric thin films crystallized by microwave radiation. *RSC Adv.* **11**, 18387–18394 (2021).

35. Y. Z. Li, J. L. Lin, Y. Bai, Y. Li, Z. D. Zhang, Z. J. Wang, Ultrahigh-energy storage properties of $(PbCa)ZrO_3$ antiferroelectric thin films via constructing a pyrochlore nanocrystalline structure. *ACS Nano* **14**, 6857–6865 (2020).

36. Q. Zhang, C. H. Jin, H. T. Xu, L. Y. Zhang, X. B. Ren, Y. Ouyang, X. J. Wang, X. J. Yue, F. Lin, Multiple-ellipse fitting method to precisely measure the positions of atomic columns in a transmission electron microscope image. *Micron* **113**, 99–104 (2018).

37. W. Weibull, A statistical distribution function of wide applicability. *J. Appl. Mech.* **73**, 293–297 (1951).

38. Y. L. Li, S. Y. Hu, Z. K. Liu, L. Q. Chen, Effect of substrate constraint on the stability and evolution of ferroelectric domain structures in thin films. *Acta Mater.* **50**, 395–411 (2002).

39. K. Xu, X. Shi, C. Shao, S. Dong, H. Huang, Design of polar boundaries enhancing negative electrocaloric performance by antiferroelectric phase-field simulations. *npj Comput. Mater.* **10**, 150 (2024).

40. Z. Liu, B.-X. Xu, Insight into perovskite antiferroelectric phases: Landau theory and phase field study. *Scr. Mater.* **186**, 136–141 (2020).

41. A. K. Tagantsev, Landau expansion for ferroelectrics: Which variable to use? *Ferroelectrics* **375**, 19–27 (2008).

42. J. Mangeri, Y. Espinal, A. Jokisaari, S. Pamir Alpay, S. Nakhmanson, O. Heinonen, Topological phase transformations and intrinsic size effects in ferroelectric nanoparticles. *Nanoscale* **9**, 1616–1624 (2017).

43. A. D. Lindsay, D. R. Gaston, C. J. Permann, J. M. Miller, D. Andrš, A. E. Slaughter, F. Kong, J. Hansel, R. W. Carlsen, C. Icenhour, L. Harbour, G. L. Giudicelli, R. H. Stogner, P. German, J. Badger, S. Biswas, L. Chapuis, C. Green, J. Hales, T. Hu, W. Jiang, Y. S. Jung, C. Matthews, Y. Miao, A. Novak, J. W. Peterson, Z. M. Prince, A. Rovinelli, S. Schunert, D. Schwen, B. W. Spencer, S. Veeraraghavan, A. Recuero, D. Yushu, Y. Wang, A. Wilkins, C. Wong, 2.0 - MOOSE: Enabling massively parallel multiphysics simulation. *SoftwareX* **20**, 101202 (2022).

44. G. Kresse, J. Furthmüller, Efficient iterative schemes for ab initio total-energy calculations using a plane-wave basis set. *Phys. Rev. B* **54**, 11169–11186 (1996).

45. G. Kresse, D. Joubert, From ultrasoft pseudopotentials to the projector augmented-wave method. *Phys. Rev. B* **59**, 1758–1775 (1999).

46. J. P. Perdew, A. Ruzsinszky, G. I. Csonka, O. A. Vydrov, G. E. Scuseria, L. A. Constantin, X. Zhou, K. Burke, Restoring the density-gradient expansion for exchange in solids and surfaces. *Phys. Rev. Lett.* **100**, 136406 (2008).

47. S. Pöykkö, D. J. Chadi, First principles study of Pb vacancies in $PbTiO_3$. *Appl. Phys. Lett.* **76**, 499–501 (2000).

48. F. F. Ge, W. D. Wu, X. M. Wang, H. P. Wang, Y. Dai, H. B. Wang, J. Shen, The first-principle calculation of structures and defect energies in tetragonal $PbTiO_3$. *Phys. B* **404**, 3814–3818 (2009).
